# DGA-Net: a dual-branch group aggregation network for liver tumor segmentation in medical images

**DOI:** 10.3389/fmedt.2025.1712952

**Published:** 2025-11-26

**Authors:** Lin Zhu, Shuyan Liu

**Affiliations:** 1 Department of Radiology, Pengshan District Hospital of Traditional Chinese Medicine, Meishan, China; 2 School of Software Engineering, Sichuan Polytechnic University, Deyang, China

**Keywords:** liver tumor segmentation, medical images, dual-branch encoder, long-range inter-pixel dependencies, spatial information

## Abstract

Hepatocellular carcinoma (HCC) is one of the most common malignant tumors worldwide. Due to its high invasiveness and poor prognosis, it ranks among the top three causes of cancer-related deaths globally. Accurate segmentation of the liver and lesion areas is crucial. It provides key support for diagnosis, surgical planning, and rehabilitation therapy. Deep learning technologies have been applied to the automatic segmentation of the liver and tumors. However, several issues remain, such as insufficient utilization of inter-pixel relationships, lack of refined processing after fusing high-level and low-level features, and high computational costs. To address insufficient inter-pixel modeling and high parameter costs, we propose DGA-Net (Dual-branch Group Aggregation Network for Liver Tumor Segmentation in Medical Images), a dual-branch architecture that includes two main components, i.e., a dual-branch encoder and a decoder with a specific module. The dual-branch encoder consists of the Fourier Spectral Learning Multi-Scale Fusion (FSMF) branch and the Multi-Axis Aggregation Hadamard Attention (MAHA) branch. The decoder is equipped with a Group Multi-Head Cross-Attention Aggregation (GMCA) module. The FSMF branch uses a Fourier network to learn amplitude and phase information. This helps capture richer features and details. The MAHA branch combines spatial information to enhance discriminative features. At the same time, it effectively reduces computational costs. The GMCA module merges features from different branches. This not only improves localization capabilities but also establishes long-range inter-pixel dependencies. We conducted experiments on the public LiTS2017 liver tumor dataset. Experiments on the public LiTS2017 liver tumor dataset show that the proposed method outperforms existing state-of-the-art approaches, achieving Dice-per-case (DPC) scores of 94.84% for liver and 69.51% for tumors, outperforming competing methods such as PVTFormer by 0.72% (liver) and 1.68% (tumor), and AGCAF-Net by 0.97% (liver) and 2.59% (tumor). We also carried out experiments on the 3DIRCADb dataset. The method still delivers excellent results, which highlights its strong generalization ability.

## 1 Introduction

The liver, the largest organ in the human abdomen, exhibits intricate anatomical structures and vascular distributions. It undertakes essential physiological functions, including metabolism, bile secretion and excretion, detoxification, hematopoiesis, and nutrient storage. Despite its remarkable regenerative capabilities, the liver remains vulnerable to numerous diseases, most notably hepatocellular carcinoma (HCC), which is characterized by elevated incidence and mortality rates. Globally, HCC ranks among the foremost causes of cancer-related mortality, with a pronounced prevalence in males. In China, it stands as the second most common cancer and the sixth leading contributor to cancer-related deaths. Many novel strategies have been established to treat various types of tumors, particularly nano-based formulations (1, 2).

Medical imaging plays an indispensable role in the diagnosis and management of liver diseases like HCC, courtesy of its high resolution, rapid acquisition speed, and cost-effectiveness. Accurate segmentation of liver and tumor regions in medical images is pivotal for precise diagnosis, rational treatment planning, and precise surgical navigation. However, this process remains predominantly manual, relying on skilled professionals to delineate target regions—a labor-intensive and subjective task that is highly susceptible to inconsistencies. Medical images are often disturbed by various noises and artifacts, including equipment electronic noise (such as Gaussian noise) or respiratory motion artifacts. These will all increase the difficulty in distinguishing tumors from normal tissues.

Advancements in computer vision and artificial intelligence have catalyzed the development of automated segmentation techniques, enabling efficient, precise, and generalizable delineation of liver and tumor regions in medical images. Despite these innovations, persistent challenges include image noise, reduced organ-tissue contrast, and irregular tumor morphologies. Liver tumor segmentation methodologies are broadly categorized into traditional image processing techniques and machine learning-based approaches. Traditional methods exploit visual features such as color and texture, employing techniques like thresholding (3), region growing (4), level sets (5, 6), and active contour models (7, 8). The emergence of enhanced computational resources has facilitated the adoption of machine learning techniques, including clustering (e.g., K-means and fuzzy C-means) (9, 10), random forests (11, 12), AdaBoost (13), and support vector machines (SVMs) (14, 15).

Deep learning has emerged as a dominant paradigm in liver tumor segmentation, with architectures such as convolutional neural networks (CNNs) (16, 17), fully convolutional networks (FCNs) (18), and U-Net (19, 20) driving significant progress. Deep learning automates feature extraction and demonstrates superior generalization. Ronneberger et al. (21) integrated the encoder-decoder into the FCN network, and introduced skip connections to solve the problem that local and global features are difficult to be balanced. UNet++ (22) performs upsampling at each layer of the encoder and also introduces dense convolution blocks to improve skip connections, enabling it to flexibly adapt to the requirements of networks with different depths. RA-UNet (23) addresses the high computational resource consumption of 3D networks by introducing a 3D hybrid residual attention-aware segmentation method, which is used to accurately extract the liver volume of interest (VOI) and segment tumors from the liver VOI. Isensee et al. (24) proposed the deep learning architecture (nnU-Net), which is specifically designed for medical image segmentation tasks, aims to provide a universal and efficient segmentation solution. Ruan et al. proposed (25) an Efficient Group Enhanced UNet (EGE-UNet), which groups input features in a lightweight manner and implements the Hadamard Product Attention mechanism on different axes, thereby extracting pathological information from multiple perspectives. Liu et al. (26) proposed a phase attention network (PA-Net) to adequately aggregate multi-phase information of CT images and improve segmentation performance for liver tumors. Wang et al. (27) proposed the attention-guided context asymmetric fusion network (AGCAF-Net), which combines attention guidance modules and context fusion modules on the basis of residual neural networks for the automatic segmentation of liver tumors. Jha et al. (28) proposed PVTFormer to improve the accuracy of liver disease diagnosis. This method is built on the pretrained Pyramid Vision Transformer (PVT v2) and incorporates advanced residual upsampling and decoder blocks. These approaches show considerable potential for supervised clinical applications, though further refinement is required for widespread adoption (29).

Although the aforementioned existing methods have achieved certain results in liver and tumor segmentation, there are still the following key issues, which form the core motivation of this research:

1. Insufficient inter-pixel relationship modeling: In liver tumor segmentation, the tumor boundary is often blurred and adjacent to normal tissues, while small lesions have sparse pixel distribution. Existing methods [e.g., vanilla U-Net (19, 20)] mainly rely on local receptive fields for feature extraction, which fail to capture long-range inter-pixel dependencies. This leads to inaccurate segmentation of tumor boundaries and easy omission of small lesions, reducing the clinical reference value of segmentation results;
2. Lack of refined processing in the fusion of high-level and low-level features: When fusing features of different levels (such as low-level edge and texture features and high-level semantic features), existing models (21–23) fail to effectively address issues like feature conflicts or information redundancy, which impairs the accurate localization of targets such as tumors;
3. High computational costs: Many advanced segmentation methods improve performance by increasing model depth or introducing complex attention mechanisms, which leads to a sharp rise in parameters. For example, some transformer-based [e.g., AGCAF-Net (27), PVTFormer (28)] methods require massive computational resources for self-attention calculations. This not only increases the burden of model training but also makes real-time inference difficult in clinical environments with limited hardware conditions, hindering practical deployment.

Based on this, we propose a Dual-branch Group Aggregation Network for Liver Tumor Segmentation in Medical Images (called DGA-Net), which can effectively address the issues of insufficient utilization of relationships between target pixels and imprecise fusion of high-level and low-level features in liver and tumor segmentation. It improves the segmentation accuracy of the liver and tumors while effectively controlling computational costs. In this method, a Fourier spectral network is primarily used to learn the amplitude and phase of spectral information, thereby enhancing feature representation in images. The Hadamard mechanism is employed to fuse spatial information, helping the network learn more discriminative features without introducing additional parameters and thus controlling computational costs. Finally, multi-head cross-attention aggregation is used to integrate feature information from different branches, establishing dependencies between pixels and improving the model's ability to localize targets. Our contributions are summarized as follows:

1. Novel dual-branch architecture: We propose DGA-Net, a dual-branch encoder-decoder model integrating Fourier spectral learning (FSMF branch) and multi-axis Hadamard attention (MAHA branch), plus a GMCA-based decoder, to balance global context and local detail capture.
2. Innovative module design: We develop three core modules—FSMF (enhances small tumor/boundary discrimination), MAHA (low-cost global pixel dependency modeling), and GMCA (improves long-range feature fusion and target localization)—each verified by ablation experiments.
3. Outstanding performance and generalization: On LiTS2017, DGA-Net outperforms 8 SOTA methods in liver/tumor segmentation [94.84%/69.51% Dice Per Case (DPC)] and shows strong cross-dataset generalization on 3DIRCADb, ranking top in multiple metrics.

## 2 Preliminaries

### 2.1 Definition 1 (Fourier spectrum)

In image processing, the Fourier spectrum reveals the orientation and strength of periodic or quas-periodic structures (30). Given a grayscale image *f*(*x*, *y*) of width *W* and height *H*, its two-dimensional discrete Fourier transform is (Equation 1):

$$F(u,v)=\sum_{x=0}^{W-1}\sum_{y=0}^{H-1}\;f(x,y)e^{-j2\pi (\frac{ux}{W}+\frac{vy}{H})}$$ (1)

Where *F*(*u*, *v*) denotes the complex amplitude at frequency coordinates (*u*, *v*). The original image is recovered by the inverse transform (Equation 2):

$$f(x,y)=\frac{1}{WH}\sum_{x=0}^{W-1}\sum_{y=0}^{H-1}F(u,v)e^{-j2\pi (\frac{ux}{W}+\frac{vy}{H})}$$ (2)

### 2.2 Definition 2 (Hadamard Product)

The Hadamard Product (31), also known as the element-wise product or Schur product, is a binary operation between matrices. Different from the standard matrix product (dot product), the Hadamard Product involves the element-wise multiplication of two matrices. Specifically, suppose there are two matrices *A* and *B* of the same order, where the elements in the *i*-th row and *j*-th column are *a_ij_* and *b_ij_* respectively. Then, the matrix *C* obtained by performing the Hadamard Product on *A* and *B* is also a matrix with the same dimension as *A* and *B*, and the element in the *i*-th row and *j*-th column of *C* is denoted as *c_ij_*. The specific formula is as follows (Equation 3):

$$c_{ij}=a_{ij}\times b_{ij}$$ (3)

## 3 Materials and methods

**Figure 1:**
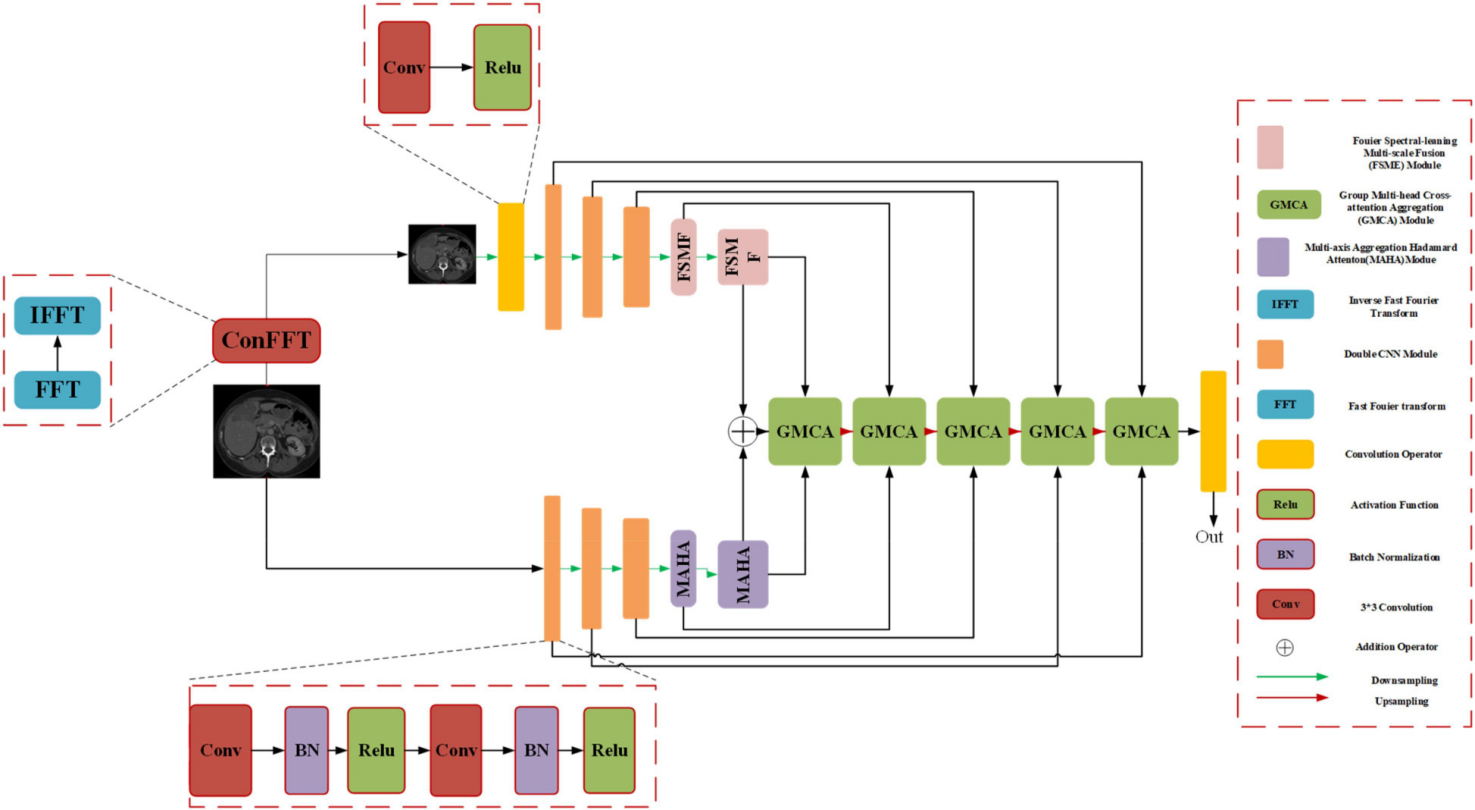
The overall architecture diagram of the DGA-Net.

We introduce an encoder-decoder architecture tailored for CT liver tumor segmentation: the Spatial and Spectral Learning Dual-Branch Aggregation Network (DGA-Net). The encoder consists of two branches: the Fourier Spectral Learning Multi-Scale Fusion (FSMF) branch and the Multi-Axis Aggregation Hadamard Attention (MAHA) branch. The FSMF branch incorporates a Fourier spectral-based network to capture the amplitude and phase of spectral information, paired with a CNN-based multi-scale fusion module to extract detailed image features. The MAHA branch applies Hadamard attention across multiple axes to each feature group, subsequently fusing them to yield richer features without excessive parameter overhead. In the decoding path, a Group Multi-Head Cross-Attention Aggregation (GMCA) module is introduced to enhance target localization precision and establish long-term dependencies among target pixels. This is accomplished by integrating multi-scale resolution features from distinct branches, supported by feature aggregation and multi-head cross-attention fusion blocks. Figure 1 depicts the overall architecture of DGA-Net, designed for segmentation of liver tumor images.

### 3.1 Overall architecture of DGA-Net

The network adopts an encoder-decoder framework, featuring two five-layer encoding branches—FSMF and MAHA—and a five-layer decoding path based on GMCA modules. In Fourier space, the amplitude component represents image brightness, while the phase component captures local detailed features. Unlike conventional spatial-spectral networks (32), DGA-Net leverages both amplitude and phase components to enhance feature representation and effectively fuse spatial information, enabling the learning of more discriminative features.

The FSMF and MAHA branches mirror the encoder structure of U-Net. In the FSMF branch, input features are processed through a Fast Fourier Transform Convolution (conFFT) block and a Convolutional Operator (conOp) block. The conFFT block applies a Fast Fourier Transform (FFT), a 1 × 1 convolution, and an Inverse FFT (IFFT), while the conOp block comprises a 3 × 3 convolution followed by ReLU activation. The output is fed into three consecutive Double Convolution (DConv) modules—each consisting of two 3 × 3 convolutions, batch normalization, and ReLU activation, followed by max-pooling—followed by two FSMF modules. These FSMF modules adaptively learn frequency-domain features from the amplitude and phase components of the FFT outputs. Similarly, the MAHA branch includes three DConv modules succeeded by two Multi-Axis Aggregation Hadamard Attention (MAHA) modules, which compute pixel weights by splitting input features, thereby reducing computational complexity.

In the decoding path, GMCA modules serve as the core units, fusing features from the FSMF branch, MAHA branch, and prior upsampled branches. Within each GMCA module, features from the fifth layer are upsampled to the second layer using bilinear interpolation to match the input image dimensions. These features are then processed by the GMCA module to extract comprehensive information through multi-head cross-attention aggregation.

### 3.2 FSMF module

Purely spatial attention networks struggle to capture subtle frequency-domain variations, so spectral learning techniques have gained traction in image analysis. In the context of medical imaging, where contrast is often low, convolution-based methods may fail to fully exploit spectral and fine-detail information. By amplifying the magnitude component via the Fourier transform, one can enhance image brightness, and the global frequency features thus revealed are highly effective at capturing target details. Integrating a Fourier-spectrum learning branch into the encoder therefore enriches feature representations without substantially increasing parameter count.

First, we review the processing of FFT. The height and width of the input image are *H* and *W* respectively. The image is first transformed into the Fourier frequency domain space using the two-dimensional Fast Fourier Transform, which can be expressed by the formula (Equation 4):

$$Y(x,y)=\sum_{h=0}^{H-1}\sum_{w=0}^{W-1}\;f(h,w)exp\left[-2\pi j\left(\frac{hx}{H}+\frac{wy}{W}\right)\right]$$ (4)

where *h* and *w* represent the coordinates transformed into the Fourier space, respectively, and *j* is the imaginary unit. Therefore, it can be rewritten into a complex number representation as (Equations 5–7):

Y(x,y)=R(Y(x,y))+jI(Y(x,y)) (5)

$$A(Y(x,y))=\sqrt{R{\left(Y(x,y)\right)}^{2}+I{\left(Y(x,y)\right)}^{2}}$$ (6)

$$\psi (Y(x,y))=\arctan\frac{I(Y(x,y))}{R(Y(x,y))}$$ (7)

where *A*[*Y*(*x*, *y*)] and *ψ*[*Y*(*x*, *y*)] represent the amplitude and phase, respectively. *R*[*Y*(*x*, *y*)] and *I*[*Y*(*x*, *y*)] are the real part and imaginary part of the Fourier function, respectively. We rewrite these two elements as follows (Equations 8, 9):

R(Y(x,y))=A(Y(x,y))×cos⁡(ψ(Y(x,y))) (8)

I(Y(x,y))=A(Y(x,y))×sin⁡(ψ(Y(x,y))) (9)

It can be seen from the above formula that the frequency-domain information covers all frequency characteristics, including short-range correlation and long-range correlation. The amplitude component *A*[*Y*(*x*, *y*)] determines the brightness information of the image, while the phase component *ψ*[*Y*(*x*, *y*)] can improve the detail information of the image. Therefore, it is very reasonable and feasible to design an automatic learning network that can adaptively learn the amplitude and spectrum components to adjust the brightness and detail information.

**Figure 2:**
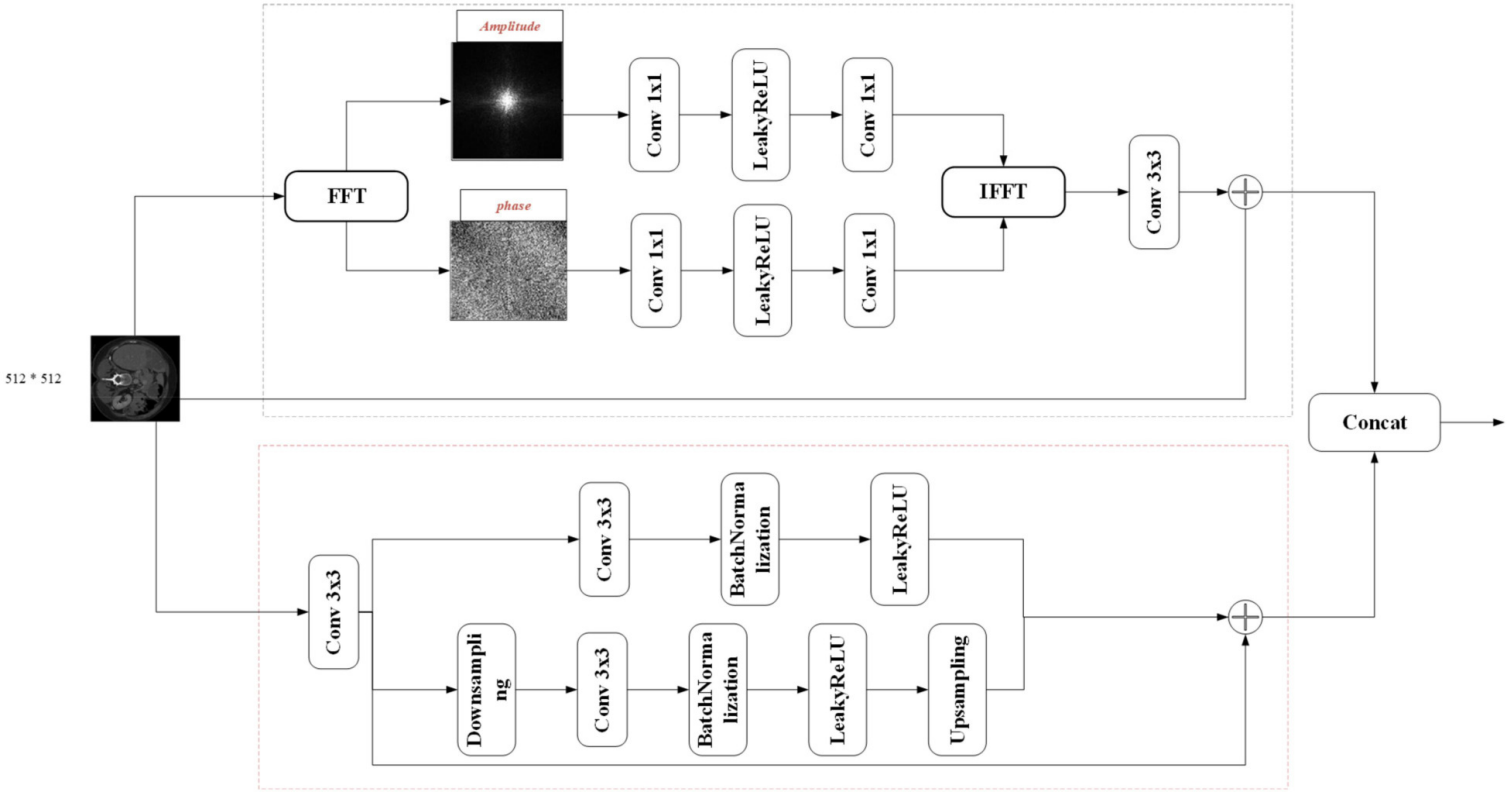
Schematic diagram of Fourier spectrum learning multi-scale fusion (FSMF).

Fourier Spectrum Learning Multi-scale Fusion (FSMF) consists of a Fourier spectrum learning module and a multi-scale residual module. The Fourier spectrum learning module is used to capture the long-term dependencies between features, and the multi-scale residual module is used to extract detailed feature information. In the Fourier spectrum learning module, FFT is first used to process the image, transforming the input feature *F_in_* into the Fourier space to obtain the amplitude component $F_{in}^{real}$ and the phase component $F_{in}^{img}$. As shown in Figure 2, for each component, this module applies an activation function (LeakyReLU) between two 1 × 1 convolutional layers, and then performs an IFFT operation on the outputs of the two components.

The spatial spectrum features are transmitted through the 3 × 3 convolutional layer and the features of the residual path, and its process formula is as follows (Equations 10–14):

$$F_{in}^{real},F_{in}^{img}=\mathrm{FFT}(F_{in})$$ (10)

$$F_{out}^{real}=conv_{1\times 1}(o_{leaky}(conv_{1\times 1}(F_{in}^{real})))$$ (11)

$$F_{out}^{img}=conv_{1\times 1}(o_{leaky}(conv_{1\times 1}(F_{in}^{img})))$$ (12)

$$F^{\;fft}=\mathrm{IFFT}(F_{out}^{real},F_{out}^{img})$$ (13)

$$F_{out}^{\;fft}=conv_{3\times 3}(F^{\;fft})+F_{in}$$ (14)

where FFT(⋅) and IFFT(⋅) represent the FFT and IFFT functions, respectively, and *o_leaky_*(⋅) represents the LeakyReLU function. In the multi-scale fusion module, in order to reduce the loss of details, three-way features are adopted and fused together through an element-wise addition operator. The first-way feature comes from the output of the convolution module, which includes a 3 × 3 convolutional layer, a batch normalization (BN) layer, and an activation (ReLU) layer. The second-way and third-way features come from the input before downsampling and the output after upsampling, respectively.

The fused feature *F^FS^* can be expressed as follows (Equations 15–17):

$$F_{in}^{ch1}=\mathrm{ReLU}(\mathrm{BN}(conv_{3\times 3}(F_{in})))$$ (15)

$$F_{in}^{ch2}=\mathrm{UP}(o_{ReLU}(\mathrm{BN}(conv_{3\times 3}(\mathrm{DN}(F_{in})))))$$ (16)

$$F^{FS}=F_{in}^{ch1}+F_{in}^{ch2}+F_{in}$$ (17)

where *o_ReLU_*(⋅) represents the ReLU activation function, UP(⋅) represents the upsampling operation, and DN(⋅) represents the downsampling operation.

### 3.3 MAHA module

Attention mechanisms have proven highly effective in image analysis, typically deriving attention weights through matrix multiplication or pointwise operations. However, existing attention modules often emphasize local pairwise interactions, constraining the ability to explore broader global feature relationships. To overcome this limitation, the DGA-Net framework introduces the Multi-Axis Aggregated Hadamard Attention (MAHA) module, inspired by Multi-Head Self-Attention (MHSA). Departing from conventional additive or multiplicative strategies, MAHA leverages the Hadamard product to integrate input features efficiently. This approach involves partitioning features into groups, applying Hadamard product-based attention along distinct axes for each group, and consolidating the outcomes through residual connections. By doing so, MAHA enhances the model's sensitivity to critical features, such as anomalies in CT images, thereby improving target region feature extraction.

**Figure 3:**
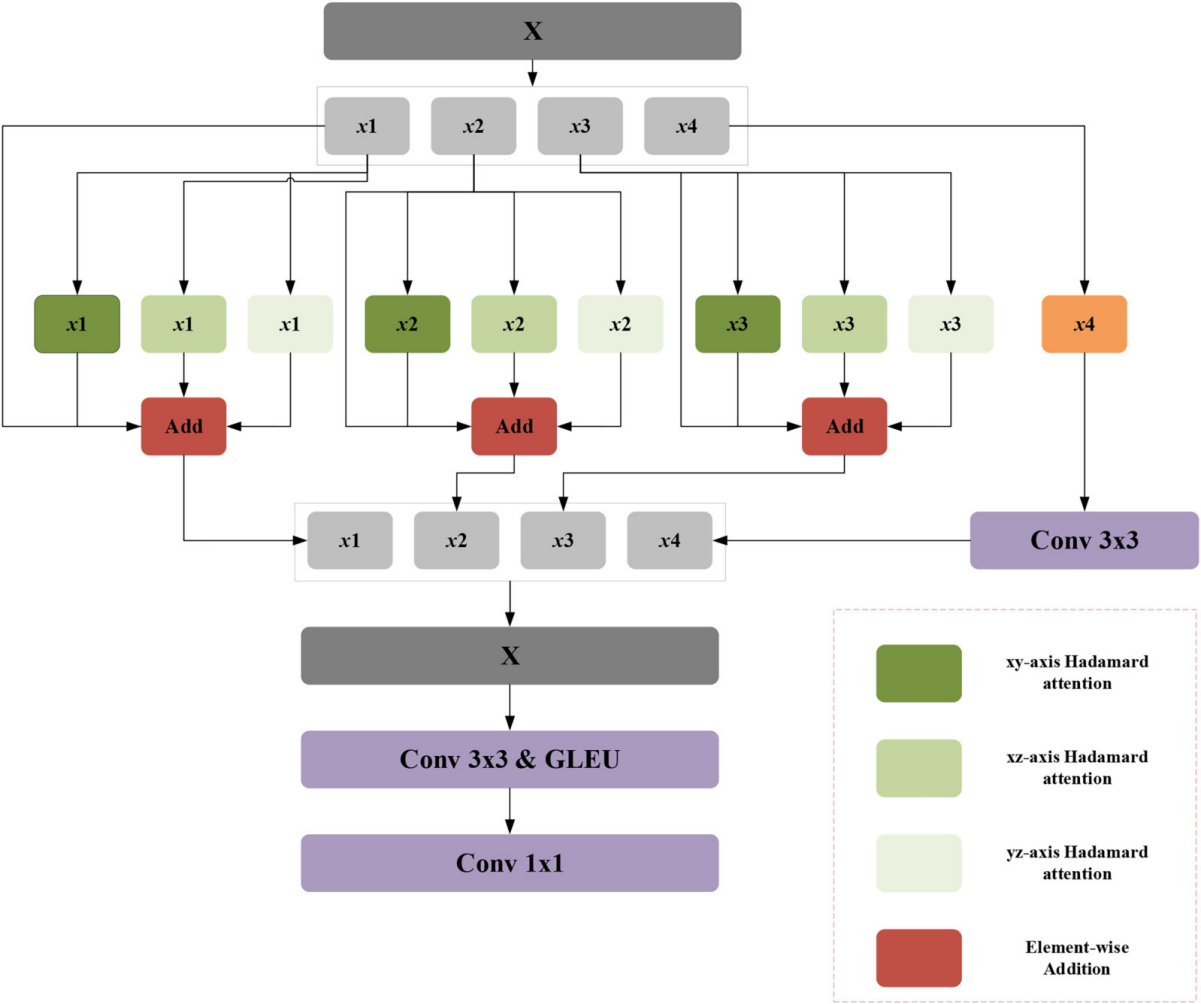
Schematic diagram of multi-axis aggregated hadamard attention (MAHA).

As depicted in Figure 3, the MAHA module initiates by dividing the input feature $F_{in}^{MH}$ along the channel dimension into four subsets: $F_{1}^{MH}$, $F_{2}^{MH}$, $F_{3}^{MH}$, $F_{4}^{MH}$. For the first three groups *i* = (1,2,3) a multi-layer perceptron (MLP) applies Hadamard product attention across three spatial axes—height-width, channel-height, and channel-width—followed by summation and residual addition to the original group feature. The fourth group undergoes processing via a 3 × 3 convolution with a subsequent residual connection. Within the Hadamard attention mechanism, weights are computed using the Hadamard product, augmented by relative position embeddings to incorporate spatial context. The processed groups are then concatenated and further refined through a 3 × 3 convolution and GELU activation, yielding the final output feature $F_{out}^{MH}$ as follows (Equations 18–20):

$$\left[F_{1}^{MH},F_{2}^{MH},F_{3}^{MH},F_{4}^{MH}]=\mathrm{chunk}(F_{in}^{MH}\right)$$ (18)

$$F_{\mathrm{ou}t_{i}}^{\mathrm{MH}}=\overset{3}{\underset{i=1}{\sum }}\mathrm{ML}P_{\mathrm{axi}s_{j}}(F_{i}^{\mathrm{MH}})+F_{i}^{\mathrm{MH}},\;j=1,2,3$$ (19)

$$F_{\mathrm{ou}t_{4}}^{\mathrm{MH}}=conv_{3\times 3}(F_{4}^{\mathrm{MH}})+F_{4}^{\mathrm{MH}}$$ (20)

where chunk(·) denotes the primary operation for feature splitting, and $axis_{j}(\cdot )$ represents the Hadamard product-based attention computation along the *j-*th axis. The Hadamard attention mechanism can be formulated as (Equation 21):

$$v_{\;j-i}=\mathrm{softmax}((q_{i}⊙k_{i})v_{\;j-i}^{k}+v_{\;j-i}^{q}(q_{j}⊙k_{j})+r_{\;j-1}^{b})$$ (21)

Here, ⊙ denotes the Hadamard attention operation. $v_{\;j-i}^{k}$ and $v_{\;j-i}^{q}$ represent the relative position embeddings of *k* and *q* respectively, while $r_{\;j-i}^{b}$ indicates the relative positional bias. Finally, the four newly generated feature groups are concatenated and passed through a 3 × 3 convolutional layer followed by a GeLU activation function. The expression is given by (Equation 22):

$$F_{\mathrm{out}}^{\mathrm{MH}}=\mathrm{GELU}(conv_{3\times 3}(concat(F_{\mathrm{ou}t_{1}}^{\mathrm{MH}},F_{\mathrm{ou}t_{2}}^{\mathrm{MH}},F_{\mathrm{ou}t_{3}}^{\mathrm{MH}},F_{\mathrm{ou}t_{4}}^{\mathrm{MH}})))$$ (22)

Conceptually, the core mechanism of MAHA is reflected in two aspects: (1) unlike traditional attention, which simplifies feature relationships into scalar weights, the Hadamard product, through element-wise multiplication of feature tensors, completely preserves the subtle correlations between each feature element, making the model more sensitive to blurred boundaries; (2) the global perspective of multi-axis aggregation: Attention is calculated along three axes—height-width, channel-height, and channel-width—breaking the limitation of a single-axis perspective. It simultaneously integrates spatial topological information and channel semantic information, achieving a dual understanding of “local details-global context.

### 3.4 GMCA module

**Figure 4:**
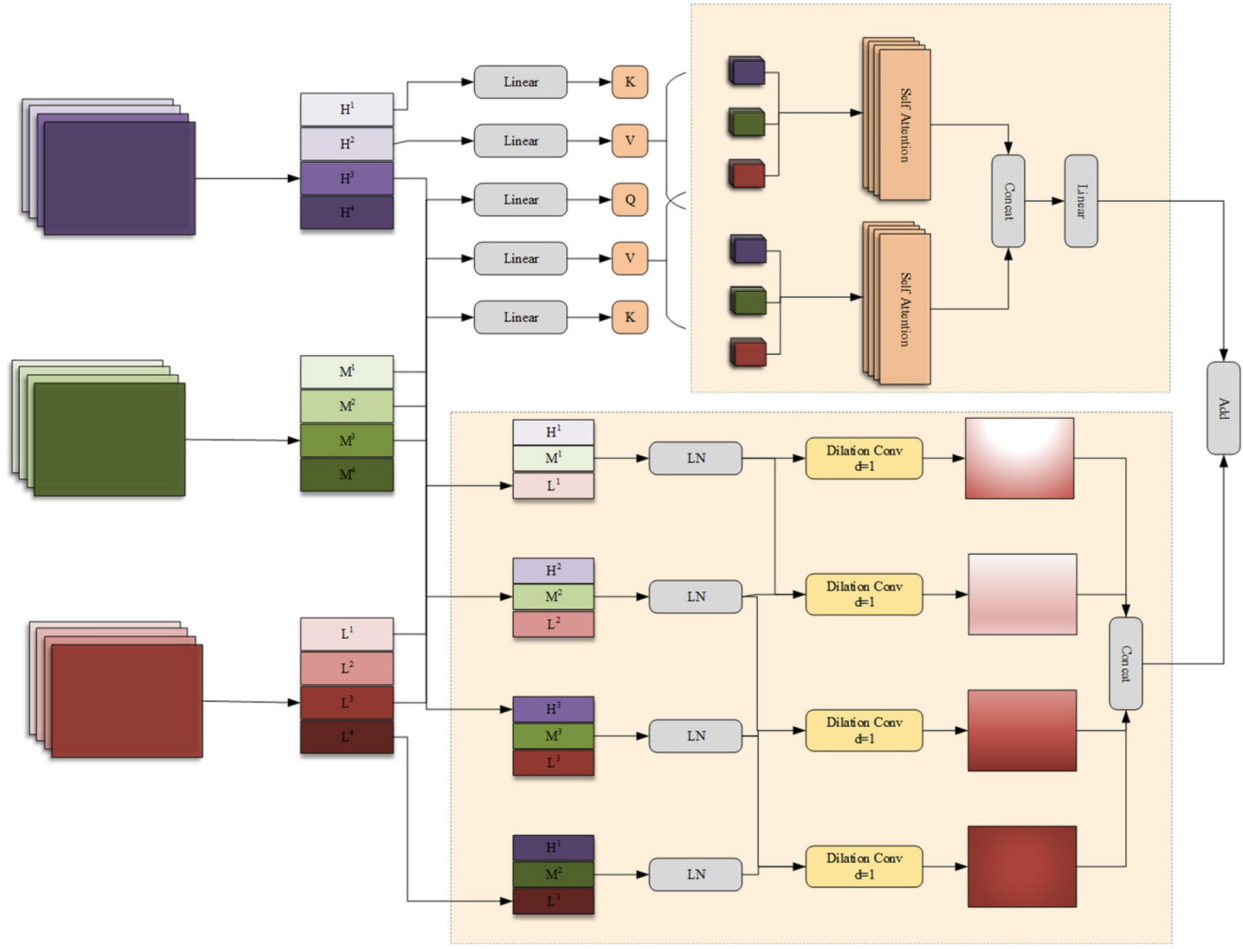
Group multi-head cross attention aggregation (GMCA) module.

In the process of encoding high-level features, the encoder may lose some low-level features, such as edges, textures, and information about small-sized targets. The network should fuse features from different branches to generate feature representations with richer information. In addition, there are significant differences in the features extracted by the encoders of the FSMF and MAHA branches for liver tumors of different sizes and dimensions in CT images. Therefore, this chapter proposes a Group-based Multi-head Attention Aggregation network (GMCA) to enrich feature representations, and its network structure is shown in Figure 4. The received features from the FSMF branch, the MAHA branch, and the output of the previous GMCA are grouped. Then, based on the feature aggregation method combined with the multi-head interactive attention mechanism, these features are aggregated to extract local information and obtain long-term dependencies.

First, we use the bilinear interpolation to upsample the output feature *F^GC^* of the previous layer in the GMCA module so that it matches the size of the input half - sized feature. The upsampled feature $F_{up}^{GC}$ and the features *F^FL^* from the FSMF branch and *F^SPHA^* from the MAHA branch are each divided into four groups. Then, the corresponding features in each group are connected $\left(F_{i}^{GC},F_{i}^{FL},F_{i}^{SPHA}\right)$ for *i* = 1,2,3,4 and layer normalization (LN) is performed. The feature $F_{i}^{Fuse}$(*i* = 1,2,3,4) of each group can be expressed as (Equations 23–27):

$$F_{up}^{GC}=\mathrm{Up}(F^{GC})$$ (23)

$$\left[F_{1}^{GC},F_{2}^{GC},F_{3}^{GC},F_{4}^{GC}]=\mathrm{chunk}(F_{up}^{GC}\right)$$ (24)

$$\left[F_{1}^{FL},F_{2}^{FL},F_{3}^{FL},F_{4}^{FL}]=\mathrm{chunk}(F^{FL}\right)$$ (25)

$$\left[F_{1}^{SHPA},F_{2}^{SHPA},F_{3}^{SHPA},F_{4}^{SHPA}]=\mathrm{chunk}(F^{SHPA}\right)$$ (26)

$$F_{i}^{Fuse}=\mathrm{LN}(\mathrm{concat}(F_{i}^{GC},F_{i}^{FL},F_{i}^{SHPA}))i=1,2,3,4$$ (27)

where concat(·) and LN(·) represent the concatenation operation and layer normalization operation, respectively.

The generated features $F_{i}^{Fuse}$(*i* = 1,2,3,4) are input in parallel into a four—branch feature aggregation block (FFAB) and a multi-head interactive attention fusion (MIAF) block. In the four-branch feature aggregation block, dilated convolutions (DilConv) with three different kernel sizes and dilation rates are used for different processing, so as to extract information of different scales. Then, by concatenating the four groups of new features $F_{i}^{DC}$(*i* = 1,2,3,4) along the channel dimension, the interaction between convolutional features with different kernel scales is realized. The specific expression of FFAB is as follows (Equations 28, 29):

$$F_{i}^{DC}=\mathrm{DilCon}v_{d}(F_{i}^{Fuse})i=1,2,3,4;d=1,2,5,7$$ (28)

$$F_{out}^{FA}=\mathrm{concat}\left(\sum_{i=1,2,3,4}F_{i}^{DC}\right)$$ (29)

In the MIAF block, the feature maps are connected to the two constructed cross-attention modules, and its implementation process is expressed as (Equations 30–34):

$$\begin{array}{rl} & Q^{FL}=\mathrm{linear}(F_{up}^{GC})W_{\mathrm{FL}}^{Q}\;\\ & K^{FL}=\mathrm{linear}(F^{FL})W_{\mathrm{FL}}^{K}\\ & V^{FL}=\mathrm{linear}(F^{FL})W_{\mathrm{FL}}^{V} \end{array}$$ (30)

$$\mathrm{MIAF}(Q^{FL})=\mathrm{softmax}\left(\frac{Q^{FL}{\left(K^{FL}\right)}^{T}}{\sqrt{D/H}}\right)V^{FL}$$ (31)

$$\begin{array}{rl} & Q^{MH}=\mathrm{linear}(F_{up}^{GC})W_{\mathrm{GC}}^{Q}\\ & K^{MH}=\mathrm{linear}(F^{MH})W_{\mathrm{GC}}^{K}\\ & V^{MH}=\mathrm{linear}(F^{MH})W_{\mathrm{GC}}^{V} \end{array}$$ (32)

$$\mathrm{MIAF}(Q^{MH})=\mathrm{softmax}\left(\frac{Q^{MH}{\left(K^{MH}\right)}^{T}}{\sqrt{D/H}}\right)V^{MH}$$ (33)

$$F_{out}^{GC}=\mathrm{MIAF}(Q^{FL})⊙\mathrm{MIAF}(Q^{MH})$$ (34)

Where linear(·) denotes a linear transformation, $W_{\mathrm{FL}}^{Q},W_{\mathrm{FL}}^{K},W_{\mathrm{FL}}^{V},W_{\mathrm{GC}}^{Q},W_{\mathrm{GC}}^{K},W_{\mathrm{GC}}^{V}$ are learnable weight matrices, *D* is the dimension of each attention head *H*, and ⊙ represents an element-wise addition or another suitable combination operation.

The GMCA module achieves deep interaction between frequency-domain features and spatial features through the collaborative design of the Four-branch Feature Aggregation Block (FFAB) and the Multi-head Interactive Attention Fusion (MIAF) block. This interaction is not a simple feature concatenation but a refined integration based on “global-local complementarity” and “dynamic weight allocation”. The features input to GMCA contain two types of core information: frequency-domain features from the FSMF branch, which are extracted based on Fourier transform and include amplitude components (reflecting the global brightness distribution of the image) and phase components (capturing local details), focusing on global context and low-frequency structural information; spatial features from the MAHA branch, which are aggregated through multi-axis Hadamard attention, focusing on spatial correlations between pixels and emphasizing local structures and high-frequency detailed information. In the FFAB, frequency-domain and spatial features first undergo multi-scale feature extraction through dilated convolutions with different dilation rates. Finally, multi-scale complementary fusion is achieved through concatenation in the channel dimension. In the MIAF, frequency-domain and spatial features realize dynamic interaction through a cross-attention mechanism. When frequency-domain features serve as queries and spatial features as keys and values, the global brightness distribution of the frequency domain guides the assignment of higher weights to local structures in spatial features that match the brightness distribution. When spatial features act as queries and frequency-domain features as keys and values, the integrity of local spatial structures corrects noise interference in frequency-domain features. This cross-attention mechanism enables the two to dynamically adjust weights based on each other's advantages, achieving a closed-loop interaction of “global constraining local and local correcting global”.

**Figure 5:**
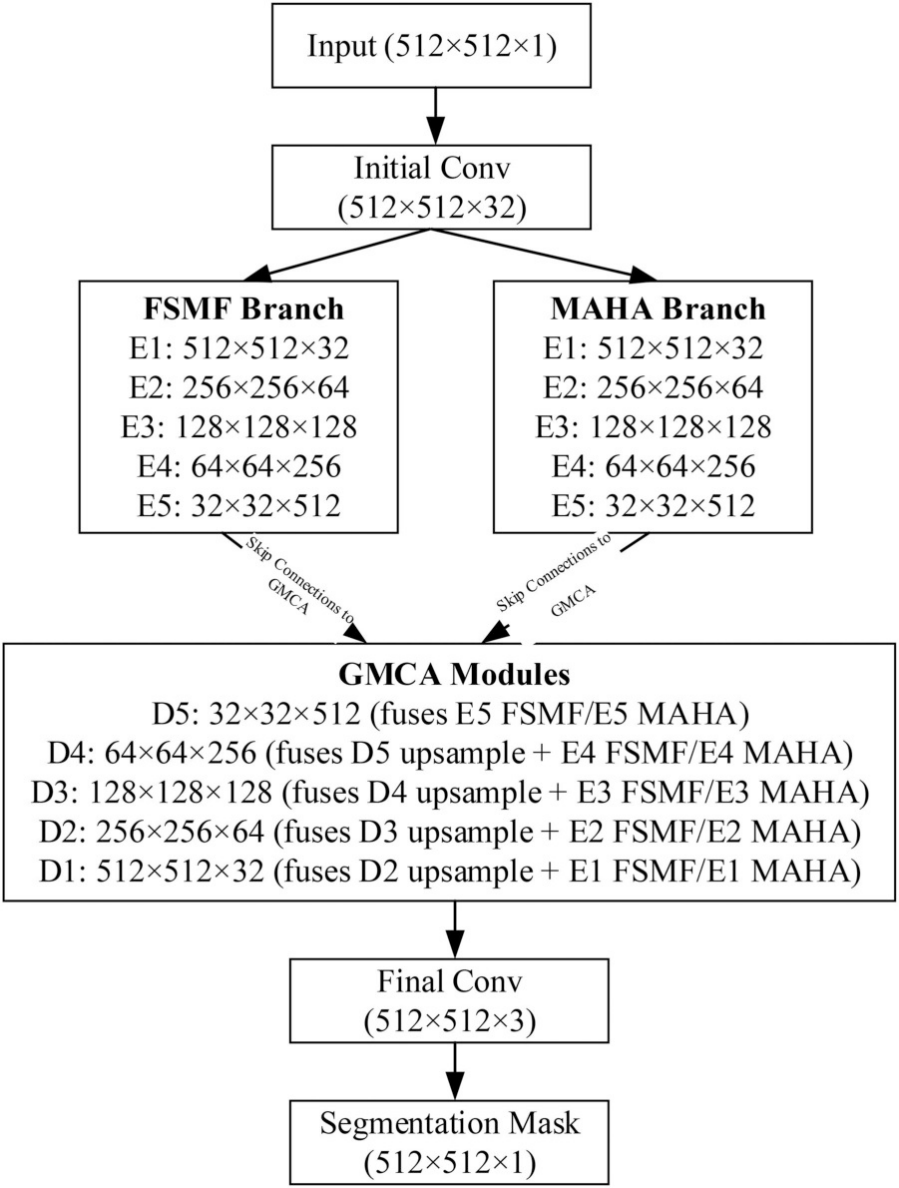
Data flow of DAG-Net.

To clarify the data flow of the DGA-Net in Figure 5, the following schematic details tensor shapes (height × width, denoted as (*H* × *W*) and channel counts (*C*) across all key stages: encoder branches (FSMF/MAHA), decoder (GMCA modules), and feature fusion. The encoder has 5 stages (E1–E5) for both FSMF and MAHA branches. Each stage (except E1) includes downsampling to capture hierarchical features. Each FSMF stage integrates a Fourier spectral-learning block (extracts amplitude/phase features) and a multi-scale residual block (fuses fine-grained details). Each MAHA stage uses multi-axis Hadamard attention (fuses spatial information across height-width, channel-height, channel-width axes) to enhance discriminative features. The decoder has 5 stages (D1–D5), each using a Group Multi-Head Cross-Attention Aggregation (GMCA) module. GMCA fuses three inputs per stage: (1) Upsampled feature map from the previous decoder stage; (2) FSMF feature map from the corresponding encoder stage (skip connection); (3) MAHA feature map from the corresponding encoder stage (skip connection).

### 3.5 Loss function

To evaluate the performance of the proposed method in aligning prediction results with ground truth labels, a minimized loss function is employed to guide the training of the prediction network. In this experiment, the composite loss function inherent to nnUNet is utilized to optimize the network's weights. This composite loss is formulated as the sum of a pixel-wise cross-entropy loss function (L_CE_) and a pixel-wise Dice loss function (L_DC_). The total loss is expressed as follows (Equation 35):

$$L=L_{CE}+L_{DC}$$ (35)

The cross-entropy loss function L_CE_ quantifies the discrepancy between the probability distributions of the true labels and the model's predictions through an information-theoretic measure (cross-entropy). This metric guides the model in adjusting its parameters to minimize the difference between predicted and actual distributions, proving particularly effective in multi-target scenarios. The computation of L_CE_ is given by (Equation 36):

$$L_{CE}=-\frac{1}{N}\sum_{n=1}^{N}y_{n}\log(p_{n})\;$$ (36)

Where y_n_ denotes the true label of the n-th image, and p_n_ represents the model's predicted probability for the n-th image. The Dice loss function L_DC_ assesses the overlap between the predicted and true labels, thereby steering the model's training to enhance segmentation accuracy.

This function is especially advantageous in addressing class imbalance challenges. It is defined in the original text as (Equation 37):

$$L_{DC}=\frac{2|X\cap Y|}{|X|+|Y|}\;$$ (37)

Where X represents the true label, Y denotes the predicted label, |X| and |Y| indicate the number of elements (pixels) in X and Y, respectively.

## 4 Results

### 4.1 Experimental settings

The network model developed is implemented using the PyTorch within the nnUNet (24) architecture. We replaced the original single-branch encoder of nnU-Net with a dual-branch structure (FSMF branch + MAHA branch). Each branch contains five encoding modules, with the channel count at each layer remaining consistent with nnU-Net. The standard convolutional modules in the nnU-Net decoder were replaced with a custom GMCA module, which is responsible for fusing dual-branch features with upsampled features. DGA-Net adheres to nnU-Net's hybrid loss concept, but the specific combination was adjusted to “Cross-Entropy loss + Dice loss” to better address the class imbalance issue in liver tumor segmentation. The raw data processing employed the default preprocessing pipeline from nnU-Net: first, the intensity values of all images were clipped to the 0.005–0.995 quantile range of the foreground voxels, and then truncated to the [−17, 201] HU interval to remove irrelevant tissues. Subsequently, the global image intensity values were normalized using the formula [*I*-mean(*I*)]/*σ*, where mean(*I*) and *σ* represent the mean and standard deviation of the image intensities, with values of 99.04 and 39.36, respectively. Finally, all spacing values from the training data were collected, and the median was selected as the target in-plane resolution spacing. All training images were resampled to 0.7676 × 0.7676 mm³. During the preprocessing, foreground regions with intensity distributions between the 0.5% and 99.5% percentiles were first extracted from the training set, followed by intensity homogenization and standard deviation operations on the extracted regions. Meanwhile, nnU-Net selected the median spacing for each axis as the target spacing, and all training sets were resampled using third-order spline interpolation. In DGA-Net, all hyperparameters except for the batch size, which was set to 4, were adopted from the default settings of nnU-Net. The maximum number of training epochs was set to 1,000. The SGD optimizer was used during training, with a learning rate of 0.01 and a polynomial weight decay set to (1-epoch/epoch_max_)⁰^.^⁹. Details regarding other hyperparameters can be found in reference (24). All experiments are conducted under uniform hardware conditions. The configuration information is described as follows: Processor (CPU) is 12th Gen Intel Core i7-12700K with a clock speed of 3.60 GHz, GPU Memory is 24 GB, Graphics Card is NVIDIA GeForce RTX 3090 (GDDR6X VRAM), OS is Ubuntu 20.04, Environment Setup is PyTorch-GPU 1.13.1, Programming Language is Python 3.10. All baselines were retrained under our unified pipeline to maximize fairness. All experimental codes and exact library versions in this study are published based on a GitHub repository^1^. The Commit Hash of the code used for final training and testing is: ce88d39.

To evaluate the segmentation performance of the model proposed in this chapter, we use the publicly available LiTS2017 dataset^2^, 3DIRCADb dataset^3^.

LiTS2017 dataset is from the MICCAI 2017 Liver Tumor Challenge. This dataset consists of 131 abdominal CT scans for training, each accompanied by original images and corresponding ground truth labels. The dataset features a diverse range of tumor sizes and quantities, including cases without cysts. For the purposes of this study, the data was partitioned into training, validation, and test sets in a 7:1:2 ratio to ensure a balanced distribution, with the cyst-free cases similarly divided. The training set contains 92 cases; The validation set contains 13 cases; The test set contains 26 cases. The label matrices are encoded with three distinct values: 0 representing the background, 1 denoting the liver, and 2 indicating tumors. The number of volume slices per scan varies (cases) from 75 to 987, with slice thicknesses ranging between 0.45 mm and 6 mm, and each slice maintains a consistent in-plane resolution of 512 × 512 pixels.

In addition, to verify the multi-faceted performance of the model, the experiment also adopted 3DIRCADb database as validation objects, which was created by the French National Institute for Computer Science and Automation (INRIA). It consists of 3D CT scans from 10 female and 10 male patients (75% of whom have liver tumors), totaling 22 sets. Since the 3DIRCADb dataset contains CT images, which are of the same category and structure as those in the LiTS2017 dataset, we directly apply the model to the 3DIRCADb dataset for testing.

### 4.2 Evaluation metrics

In this experiment, five evaluation metrics commonly used in medical image segmentation, especially in liver tumor segmentation, were adopted. These evaluation metrics (33, 34) are DPC (Dice Per Case), DG (Dice Global), VOE (Volumetric Overlap Error), RAVD (Relative Absolute Volume Difference), and ASSD (Average Symmetric Surface Distance). Suppose the real label of an image is denoted as A, and the result predicted by the network model is denoted as B. Taking the number of target categories as 1 as an example, all pixel points in A and B can only be 0 or 1, where 0 represents the background and 1 represents the target. |A| and |B| respectively represent the number of target pixels, i.e., the number of pixel points with a value of 1.

DPC is an evaluation metric based on Dice for evaluating the performance of segmentation models, which aims to assess the overall segmentation effect of different data (i.e., images of different cases). Their formula definitions are as follows (Equations 38, 39):

$$Dice(A,B)=\frac{2|A\cap B|}{\left|A|+|B\right|}$$ (38)

$$DPC=\frac{\sum_{i=1}^{N}Dice_{i}}{N}$$ (39)

DG is used to measure the average performance of the segmentation algorithm on the entire dataset. Its formula definition is as follows (Equation 40):

$$DG=\frac{2\times \sum_{i=1}^{N}\left|A_{i}\cap B_{i}\right|}{\sum_{i=1}^{N}\left|A_{i}\right|+\sum_{i}^{N}\left|B_{i}\right|}$$ (40)

VOE is an evaluation metric that evaluates the accuracy of image segmentation by measuring the non-overlapping parts between the volume of the predicted result and that of the real label. Its formula definition is as follows (Equation 41):

$$VOE(A,B)=1-\frac{A\cap B}{A\cup B}$$ (41)

RAVD is not as common as several other evaluation metrics, but it can intuitively measure the volume difference between the real label and the predicted label. It takes the ratio of the absolute volume difference between the real label and the predicted result to the volume of the real label as the result. Its formula definition is as follows (Equation 42):

$$RAVD(A,B)=\frac{\left|A|-|B\right|}{\left|A\right|}$$ (42)

ASSD serves to gauge the surface distance between predicted outcomes and actual labels. Its formula definition is as follows [where d(⋅) represents the distance between two points] (Equations 43, 44):

$$ASD(A,B)=\sum_{a}^{A}\frac{\min_{b\in B}d(a,b)}{\left|A\right|}$$ (43)

$$ASSD(A,B)=\frac{ASD(A,B)+ASD(B,A)}{2}$$ (44)

HD95 quantifies the maximum distance between 95% of corresponding boundary points in the predicted and ground-truth regions, with smaller values indicating better boundary alignment. The formula is as follows (Equation 45):

95%HD(A,B)=quantile(D,0.95) (45)

Where D represents the merged distance set, A and B denote different point sets, and HD(·) denotes the Hausdorff distance function.

### 4.3 Comparative experiments

In the following experiments, simultaneous single-stage multi-class segmentation of both the liver and tumors was conducted on the LiTS2017 dataset. To quantitatively assess the segmentation performance of the proposed DGA-Net, it was compared against nine models: U-Net (21), UNet++ (22), RA-UNet (23), nnUNet (24), EGE-UNet (25), PA-Net (26), AGCAF-Net (27) and PVTFormer (28). Additionally, the segmentation results of DGA-Net were discussed in relation to state-of-the-art (SOTA) 2D models. Given the distinct characteristics and segmentation challenges posed by the liver and tumors, the results for each were analyzed separately, with a particular focus on tumor segmentation.

#### 4.3.1 Analysis of liver segmentation results on LiTS2017

Table 1 presents a quantitative comparison of various methods for liver segmentation on the LiTS2017 dataset against SOTA approaches. Looking at the parameter count [Para. (M)], our proposed DGA-Net has 13.91M parameters, which is relatively low compared to many other methods like nnUNet (126.43M) and PVTFormer (45.05M). This indicates that DGA-Net is more parameter-efficient, potentially requiring less computational resources. In terms of DPC, DGA-Net achieves 0.9484, which is close to the highest value of 0.9541 attained by PVTFormer. For DG, DGA-Net's 0.9483 is competitive with other top-performing methods. The VOE of DGA-Net is 0.1053, lying within a favorable range among the compared methods. Most importantly, DGA-Net achieves the best performance in RAVD with a value of 0.0393 and the lowest ASSD at 2.80. These two metrics are crucial for assessing the accuracy of volume measurement and surface distance, respectively. To ensure the reliability of the experiment, we repeated each group of experiments three times under the same conditions and took the average value. Among them, the values in parentheses are standard deviations, and those in square brackets are 95% confidence intervals (95%CI).

**Table 1:** Quantitative comparison of livers on LiTS2017 with SOTA method.

Method	Para.(M)	DPC	DG	VOE	RAVD	ASSD
UNet (21)	34.53	0.9394 (0.0085) [0.9187,0.9601]	0.9382 (0.0085) [0.9175,0.9589]	0.1134 (0.0032) [0.1055,0.1213]	0.0663 (0.0125) [0.0355,0.0971]	6.34 (1.32) [3.07,9.60]
UNet++ (22)	47.18	0.9405 (0.0096) [0.9168,0.9642]	0.9398 (0.0072) [0.9220,0.9576]	0.1111 (0.0037) [0.1020,0.1202]	0.0623 (0.0131) [0.0299,0.0947]	6.41 (1.11) [3.66,9.15]
RA-UNet (23)	34.88	0.9472 (0.0088) [0.9255,0.9689]	0.9472 (0.0067) [0.9306,0.9638]	0.0994 (0.0043) [0.0888,0.1100]	0.0566 (0.0094) [0.0334,0.0798]	4.07 (0.97) [1.67,6.46]
nnUNet (24)	126.43	0.9523 (0.0077) [0.9333,0.9713]	**0.9541 (0.0042)** [0.9437,0.964]	**0.0905 (0.0022)** [0.0851,0.0959]	0.0427 (0.0079) [0.0232,0.0622]	**2.47 (0.82)** [0.44,4.50]
EGE-UNet (25)	**0**.**05**	0.9048 (0.0102) [0.8796,0.9300]	0.9041 (0.0113) [0.8762,0.9320]	0.1713 (0.0131) [0.1389,0.2037]	0.0792 (0.0147) [0.0429,0.1155]	8.05 (0.91) [5.79,10.3]
PA-Net (26)	39.72	0.9468 (0.0087) [0.9253,0.9683]	0.9472 (0.0077) [0.9282,0.9662]	0.1003 (0.0035) [0.0917,0.1089]	0.0571 (0.0061) [0.0420,0.0722]	4.18 (0.77) [2.27,6.08]
AGCAF-Net (27)	42.88	0.9437 (0.0101) [0.9187,0.9687]	0.9482 (0.0089) [0.9263,0.9701]	0.1072 (0.0047) [0.0956,0.1188]	0.0472 (0.0041) [0.0371,0.0573]	3.54 (0.67) [1.88,5.19]
PVTFormer (28)	45.05	**0.9541 (0.0038)** [0.9447,0.963]	0.9497 (0.0043) [0.9391,0.9603]	0.1088 (0.0052) [0.0959,0.1217]	0.0418 (0.0064) [0.0260,0.0576]	3.11 (0.54) [1.77,4.44]
**Ours**	13.91	0.9484 (0.0059) [0.9338,0.9630]	0.9483 (0.0051) [0.9357,0.9609]	0.1053 (0.0039) [0.0957,0.1149]	**0.0393 (0.0041)** [0.0292,0.049]	2.80(0.43) [1.73,3.86]

Bold values represent the best mean value.

Some models’ attention modules (such as AGCAF-Net) calculate weights through multi-layer fully connected networks. Although this enhances feature interaction, it introduces excessive parameters, which easily leads to overfitting when processing high-resolution CT slices and results in poor generalization ability for rare tumor subtypes in training data. Standard self-attention has a time complexity of O(*L*^2^·*d*) (*L* = number of pixels, *d* = feature dimension) due to *Q*-*K* matrix multiplication, which is computationally expensive for high-resolution medical images. Hadamard attention replaces this with element-wise multiplication [*O*(*L*·*d*) complexity] and adds relative position embeddings (lightweight parameter addition) instead of learnable *Q*/*K*/*V* projection matrices. Unlike the MAHA module of DGA-Net, which reduces parameters through “feature grouping + residual paths”, these modules struggle to balance feature richness and processing efficiency with limited computing resources, ultimately affecting segmentation performance. Overall, DGA-Net demonstrates a good balance between parameter efficiency and segmentation accuracy, outperforming other methods in key metrics related to volume and surface distance, and showing competitive performance in dice-based metrics.

In additional, our model exhibits prominent advantages with excellent performance balance and strong practicality: it features a lightweight design with only 13.91M parameters, achieving efficient inference while maintaining high segmentation accuracy—with an inference time of 0.082 s per 512 × 512 slice and 35.4 s per typical LiTS volume (average 432 slices, 432 × 512 × 512) including preprocessing and postprocessing, accompanied by computational metrics of 8.72 × 10⁹ FLOPs and 4.36 × 10⁹ MACs per slice, as well as a peak GPU memory usage of 4.2GB during inference. Notably, the FSMF branch and GMCA module effectively preserve volume-level spatial consistency in slice-level inference to avoid local mis-segmentation, and DGA-Net has the 35.4-second volume inference latency.

#### 4.3.2 Analysis of tumor segmentation results on LiTS2017

Table 2 conducts a quantitative comparison of different methods for tumor segmentation on the LiTS2017 dataset with SOTA approaches. We also show paired two-tailed t-tests (*α*=0.05) with DGA-Net(Ours), and the critical value of |*t*| is 4.303. In terms of DPC, our method attains a value of 0.6951, which is the highest among all the compared methods. For DG, our method reaches 0.8182, surpassing other methods and indicating a better overall segmentation consistency. Regarding VOE, our method has a value of 0.4395, the lowest in the table, suggesting a more accurate volume overlap between the segmented tumor and the ground truth. For RAVD, our method achieves 0.3861, which is also the best, meaning the volume difference between the segmented tumor and the actual tumor is minimized. In terms of ASSD, our method gets 10.80, the lowest among all, implying that the surface distance between the segmented tumor and the real tumor is the smallest, reflecting high precision in surface segmentation.

**Table 2:** Quantitative comparison of tumors on LiTS2017 with SOTA method.

Method	DPC	DG	VOE	RAVD	ASSD
UNet (21)	0.6408 (0.0324) [0.5606,0.7210]	0.7761 (0.0217) [0.7224,0.8298]	0.4963 (0.0178) [0.4523,0.5403]	0.7483 (0.0307) [0.6723,0.8243]	19.76 (1.43) [16.22,23.29]
	*t* = 2.92, NS	*t* = 3.36, NS	*t* = −5.53, S	*t* = −20.67, S	*t* = −11.02, S
UNet++ (22)	0.6651 (0.0387) [0.5694,0.7608]	0.7908 (0.0254) [0.7280,0.8536]	0.4753 (0.0164) [0.4347,0.5159]	0.6057 (0.0357) [0.5174,0.6940]	16.17 (1.88) [11.51,20.82]
	*t* = 1.14, NS	*t* = 1.85, NS	*t* = −3.77, NS	*t* = −10.83, S	*t* = −4.98, S
RA-UNet (23)	0.6866 (0.0327) [0.6056,0.7676]	0.7983 (0.0233) [0.7406,0.8560]	0.4486 (0.0169) [0.4068,0.4904]	0.5234 (0.0368) [0.4323,0.6145]	11.50 (0.59) [10.03,12.96]
	*t* = 0.45, NS	*t* = 1.47, NS	*t* = −0.93, NS	*t* = −6.59, S	*t* = −2.11, NS
nnUNet (24)	0.6427 (0.0254) [0.5799,0.7055]	0.7689 (0.0244) [0.7085,0.8293]	0.4996 (0.0179) [0.4554,0.5438]	0.8359 (0.0291) [0.7639,0.9079]	17.95 (1.32) [14.68,21.21]
	*t* = 3.59, S	*t* = 3.49, S	*t* = −5.83, S	*t* = −27.23, S	*t* = −9.34, S
EGE-UNet (25)	0.4875 (0.0289) [0.4159,0.5591]	0.7001 (0.0325) [0.6196,0.7806]	0.6524 (0.0169) [0.6106,0.6942]	1.8548 (0.0402) [1.7553,1.9543]	35.93 (2.54) [29.64,42.21]
	*t* = 12.37, S	*t* = 6.18, S	*t* = −21, S	*t* = −63.24, S	*t* = −17.03, S
PA-Net (26)	0.6719 (0.0297) [0.5984,0.7454]	0.8085 (0.0177) [0.7647,0.8523]	0.4493 (0.0181) [0.4045,0.4941]	0.4903 (0.0284) [0.4200,0.5606]	12.37 (1.11) [9.62,15.11]
	*t* = 1.38, NS	*t* = 0.95, NS	*t* = −0.94, NS	*t* = −6.38, S	*t* = −2.43, NS
AGCAF-Net (27)	0.6841 (0.0228) [0.6277,0.7405]	0.7904 (0.0217) [0.7367,0.8441]	0.4501 (0.0151) [0.4128,0.4874]	0.4000 (0.0298) [0.3263,0.4737]	11.20 (0.20) [10.70,11.69]
	*t* = 0.83, NS	*t* = 2.24, NS	*t* = −1.24, NS	*t* = −0.83, NS	*t* = −3.46, NS
PVTFormer (28)	0.6878 (0.0239) [0.6287,0.7469]	0.8020 (0.0197) [0.7533,0.8507]	0.4420 (0.0143) [0.4066,0.4774]	0.3950 (0.0287) [0.3240,0.4660]	11.00 (0.25) [10.38,11.61]
	*t* = 0.54, NS	*t* = 1.43, NS	*t* = −0.3, NS	*t* = −0.55, NS	*t* = −1.39, NS
**Ours**	**0.6951 (0.0203)** [0.6449,0.7453]	**0.8182 (0.0180)** [0.7737,0.8627]	**0.4395 (0.0137)** [0.4056,0.4734]	**0.3861 (0.0271)** [0.3191,0.4531]	**10.80(0.15)** [10.42,11.17]

Bold values represent the best mean value.

**Figure 6:**
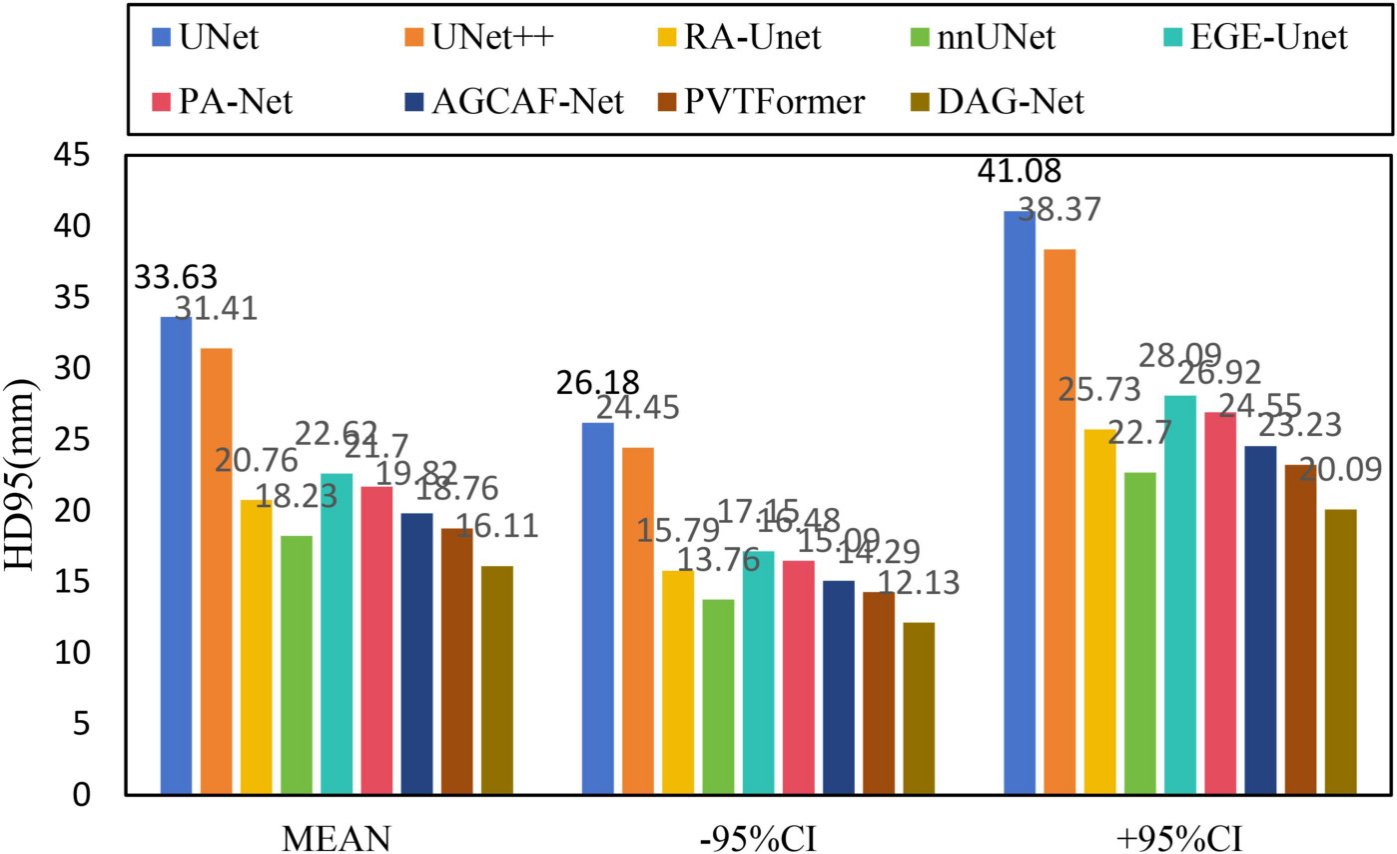
HD95 of each model on LiTS2017.

Figure 6 shows the experimental results of HD95 on LiTS2017. The experimental environment and settings are consistent, and each model is run three times, with the average value taken. And we report 95%CI. DGA-Net has the lowest HD95, indicating that the spatial difference between the tumor segmentation result and the ground truth is the smallest, and the segmentation accuracy is the highest.

Existing models (such as RA-UNet, AGCAF-Net) mostly adopt single-dimension attention weight assignment (for example, only based on spatial position or channel dimension), and the fusion of high-and low-level features relies on simple concatenation or element-wise addition, lacking refined modeling of feature correlation. Their attention mechanisms mostly focus on the “salient screening” of local features, but unlike the MAHA module of DGA—Net, they do not establish global pixel dependencies in dimensions such as height-width and channel-height through multi-axis Hadamard product, resulting in insufficient capture of pixel—level correlations for irregular tumors with blurred edges and distorted shapes. Overall, our method outperforms other SOTA methods in all these key metrics, demonstrating superior performance in tumor segmentation on the LiTS2017 dataset, both in terms of overall and phase-consistent segmentation, as well as in the accuracy of volume and surface representation.

**Figure 7:**
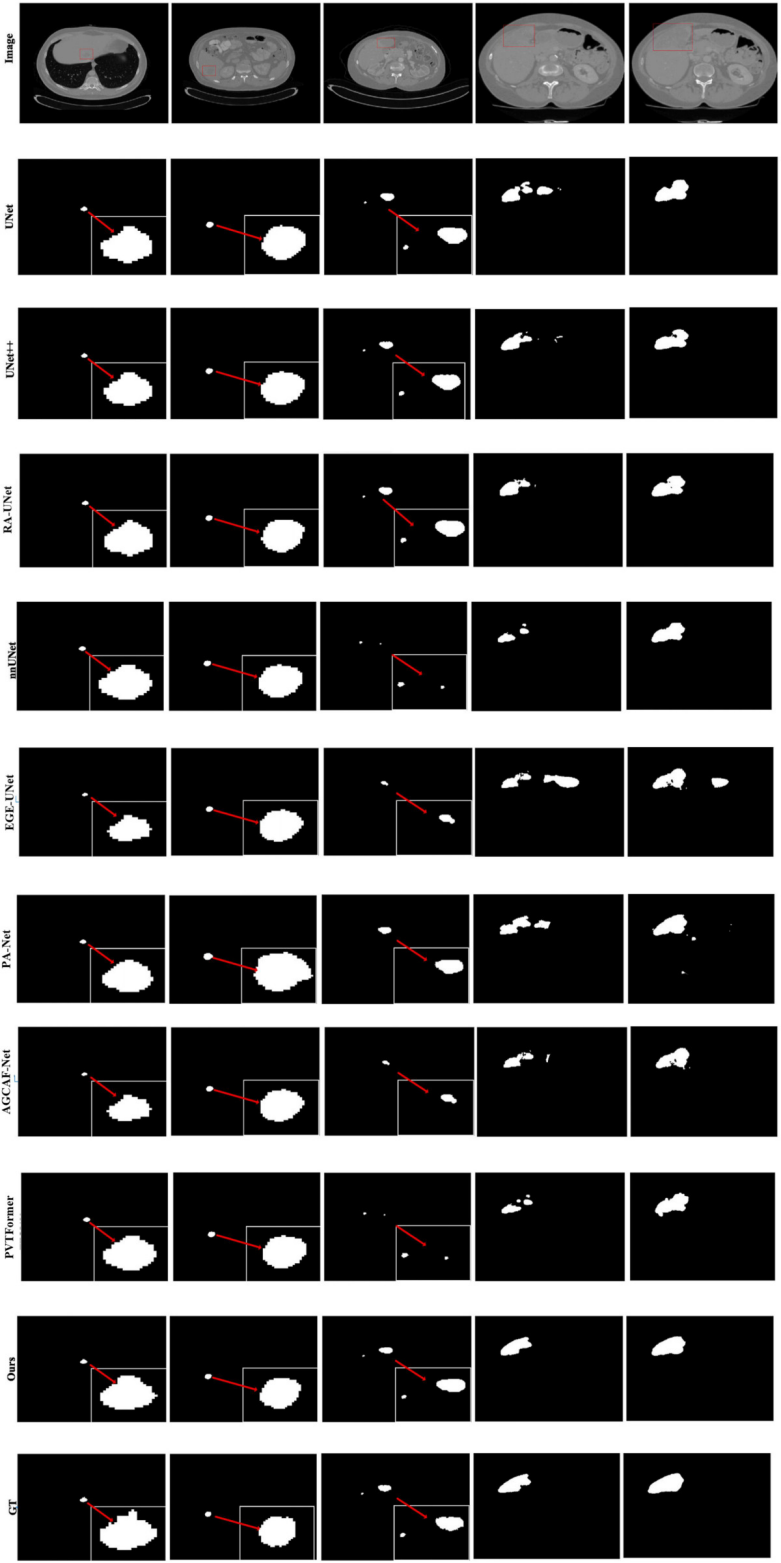
Tumor segmentation results of each model on LiTS2017.

Given the generally high performance and minimal differences in liver segmentation across models, only tumor segmentation results are visually presented to highlight the distinct performance advantages. Figure 7 provides a visual comparison of tumor segmentation outcomes from various models. Compared to other approaches, DGA-Net exhibits superior performance in handling small tumor regions and capturing tumor boundaries.

In the first and second columns of the figure, DGA-Net's segmentation of small tumors closely matches the ground truth in terms of size and extent. In the third column, where tumors are less distinct or resemble surrounding normal tissues, DGA-Net demonstrates robust localization capabilities, outperforming most other methods. Additionally, in the fourth and fifth rows, DGA-Net maintains continuity in segmentation results, showcasing its competitive edge in accurately detecting irregular large liver regions, unlike some models that produce fragmented outputs. In summary, the proposed DGA-Net effectively addresses the challenges inherent in liver tumor segmentation. The experimental results confirm that DGA-Net not only maintains high performance in liver segmentation but also achieves superior results in tumor segmentation compared to existing methods.

### 4.4 Ablation experiments

To comprehensively evaluate the effectiveness of individual modules and the overall rationality of the proposed DGA-Net model, this section presents a series of ablation experiments. All ablation experiments use identical random seeds (fixed to 42 for PyTorch/NumPy/Python) and computational budgets (1,000 epochs, batch size = 4, identical optimizer) to ensure that variability only arises from architectural differences, not setup discrepancies. All settings are consistent with those of the comparative experiments.

#### 4.4.1 Effectiveness of each module

In this section, the impact of each component in the proposed model is evaluated through module ablation, including 6 ablation experiments such as the four modules of ConvFFT, FSMF, MAHA and GMCA, as well as connection methods and branch ablation. During the experiment, the same training parameters as those in the DGA-Net model experiment are still maintained.

Table 3 shows the results of the ablation experiments for each module on the liver, with the optimal value of each indicator in the table highlighted in bold. It is not difficult to see from the table that, except for the ConvFFT module, the addition of each module effectively improves the model's segmentation effect on the liver region. In particular, the addition of GMCA directly increases the DPC by nearly 2%. However, the addition of ConvFFT causes a slight decrease in DPC, indicating that ConvFFT has a limited effect on improving the brightness of the liver region and does not play the ideal role.

**Table 3:** Results of ablation experiments of each module on the liver.

Method	DPC	DG	VOE	RAVD	ASSD
w/o FSMF	0.9464 (0.0060) [0.9315, 0.9613]	0.9483 (0.0043) [0.9377, 0.9589]	0.1002 (0.0045) [0.0893,0.1111]	0.0506 (0.0049) [0.0386,0.0626]	3.19 (0.41) [2.200, 4.180]
w/o ConvFFT	**0.9505 (0.0064)** [0.9349, 0.9661]	**0.9517 (0.0059)** [0.9370, 0.9664]	**0.0920 (0.0041)** [0.0818,0.1022]	**0.0378 (0.0048)** [0.0258,0.0498]	4.47 (0.43) [3.400, 5.540]
w/o GMCA	0.9293 (0.0061) [0.9145, 0.9441]	0.9384 (0.0057) [0.9245, 0.9523]	0.1285 (0.0041) [0.1183,0.1387]	0.0578 (0.0051) [0.0453,0.0703]	8.46 (0.44) [7.385, 9.545]
w/o MAHA	0.9406 (0.0049) [0.9286, 0.9526]	0.9407 (0.0051) [0.9282, 0.9532]	0.1100 (0.0038) [0.1007,0.1193]	0.0544 (0.0043) [0.0438,0.0650]	8.25 (0.39) [7.297, 9.203]
w/o One-Branch	0.9487 (0.0051) [0.9362, 0.9612]	0.9493 (0.0054) [0.9361, 0.9625]	0.0971 (0.0047) [0.0856,0.1086]	0.0548 (0.0045) [0.0439,0.0657]	5.64 (0.40) [4.662, 6.618]
w/o Add(concat)	0.9405 (0.0057) [0.9266, 0.9544]	0.9404 (0.0049) [0.9284, 0.9524]	0.1039 (0.0042) [0.0936,0.1142]	0.0422 (0.0038) [0.0329,0.0515]	5.53 (0.42) [4.501, 6.559]
**Ours**	0.9484 (0.0059) [0.9338,0.9630]	0.9483 (0.0051) [0.9357,0.9609]	0.1053 (0.0039) [0.0957,0.1149]	0.0393 (0.0041) [0.0292,0.049]	**2.80(0.43)** [1.73,3.86]

Bold values represent the best mean value.

The FSMF and MAHA modules optimize feature representation through the complementarity of frequency-domain and spatial-domain features as well as the synergy of global and local information. Their interaction is reflected in three aspects: First, the complementary filling of frequency-domain and spatial-domain features. FSMF provides a discriminative benchmark, while MAHA optimizes spatial-domain features and reduces parameters, forming a “global constraint + local calibration” mechanism. Second, the synergistic enhancement of global and local features. FSMF fuses multi-scale frequency-domain features but lacks spatial modeling; MAHA strengthens pixel correlations and embeds them into the global background. The two improve efficiency and avoid “pseudo-features”. Third, the mutual reinforcement of feature robustness. FSMF reduces noise through frequency-domain filtering, and MAHA further filters residual noise and strengthens effective features. Their synergistic effect is crucial for high segmentation accuracy in complex scenarios. In summary, through these three interactions, the two modules construct high-quality features, laying the foundation for subsequent aggregation by the GMCA module and ultimately improving liver tumor segmentation performance.

Table 4 presents the results of the ablation experiments for each module on tumors, with the optimal value of each indicator in the table highlighted in bold. It can be clearly seen from the first row that the addition of the FSMF module significantly helped the model improve DPC by 2.44% and DG by 0.42%. Results of the second row show that unlike its ineffective performance in liver segmentation, ConvFFT greatly enhanced the performance of the DGA-Net in this chapter, with DPC increasing by 3.6% and DG rising by an impressive 20.67%. In the third row, the GMCA module increased DPC and DG by 8.19% and 4.57% respectively. Regarding the MAHA module in the fourth row, it improved the model's DPC by 1.68% and DG by 0.93%. The fifth row shows the experiment with the FSMF branch removed, and the results indicate that a single spatial branch cannot outperform the combination of dual branches, while the construction of dual branches has improved the model overall, with DPC increasing by 2.82% and DG by 13.88%. Finally, ablation was also performed on the fusion method within the GMCA module, comparing the impact of pixel-wise addition and concatenation operations on the model. It was found that pixel-wise addition not only improved segmentation performance but also reduced costs.

**Table 4:** Results of ablation experiments of each module on tumors.

Method	DPC	DG	VOE	RAVD	ASSD
w/o FSMF	0.6707 (0.0195) [0.6229, 0.7185]	0.8136 (0.0179) [0.7697,0.8575]	0.4427 (0.0131) [0.4106,0.4748]	0.7484 (0.0270) [0.6823, 0.8145]	16.39 (0.14) [16.047, 16.733]
w/o ConvFFT	0.6591 (0.0201) [0.6097, 0.7085]	0.6115 (0.0191) [0.5647, 0.6583]	0.4822 (0.0147) [0.4462, 0.5182]	0.4838 (0.0271) [0.4174, 0.5492]	12.64 (0.13) [12.322, 12.958]
w/o GMCA	0.6132 (0.0207) [0.5626,0.6638]	0.7725 (0.0189) [0.7262,0.8188]	0.5225 (0.0144) [0.4872, 0.5578]	0.5838 (0.0265) [0.5190, 0.6486]	22.17 (0.15) [21.803, 22.537]
w/o MAHA	0.6783 (0.0215) [0.6256,0.7310]	0.8089 (0.0184) [0.7638, 0.8540]	0.4467 (0.0139) [0.4127, 0.4807]	0.4155 (0.0268) [0.3500, 0.4810]	11.69 (0.17) [11.274, 12.106]
w/o One-Branch	0.6669 (0.0210) [0.6155,0.7183]	0.6794 (0.0173) [0.6370, 0.7218]	0.4739 (0.0131) [0.4418, 0.5060]	0.4929 (0.0284) [0.4233, 0.5625]	25.09 (0.18) [24.650, 25.530]
w/o Add (concat)	0.6839 (0.0197) [0.6356,0.7322]	0.7257 (0.0175) [0.6828, 0.7686]	0.4593 (0.0135) [0.4262, 0.4924]	0.4559 (0.0279) [0.3876, 0.5242]	15.59 (0.16) [15.200, 15.980]
**Ours**	**0.6951 (0.0203)** [0.6453,0.7449]	**0.8182 (0.0180)** [0.7741, 0.8623]	**0.4395 (0.0137)** [0.4059, 0.4731]	**0.3861 (0.0271)** [0.3197, 0.4525]	**10.80(0.15)** [10.433, 11.167]

Bold values represent the best mean value.

**Figure 8:**
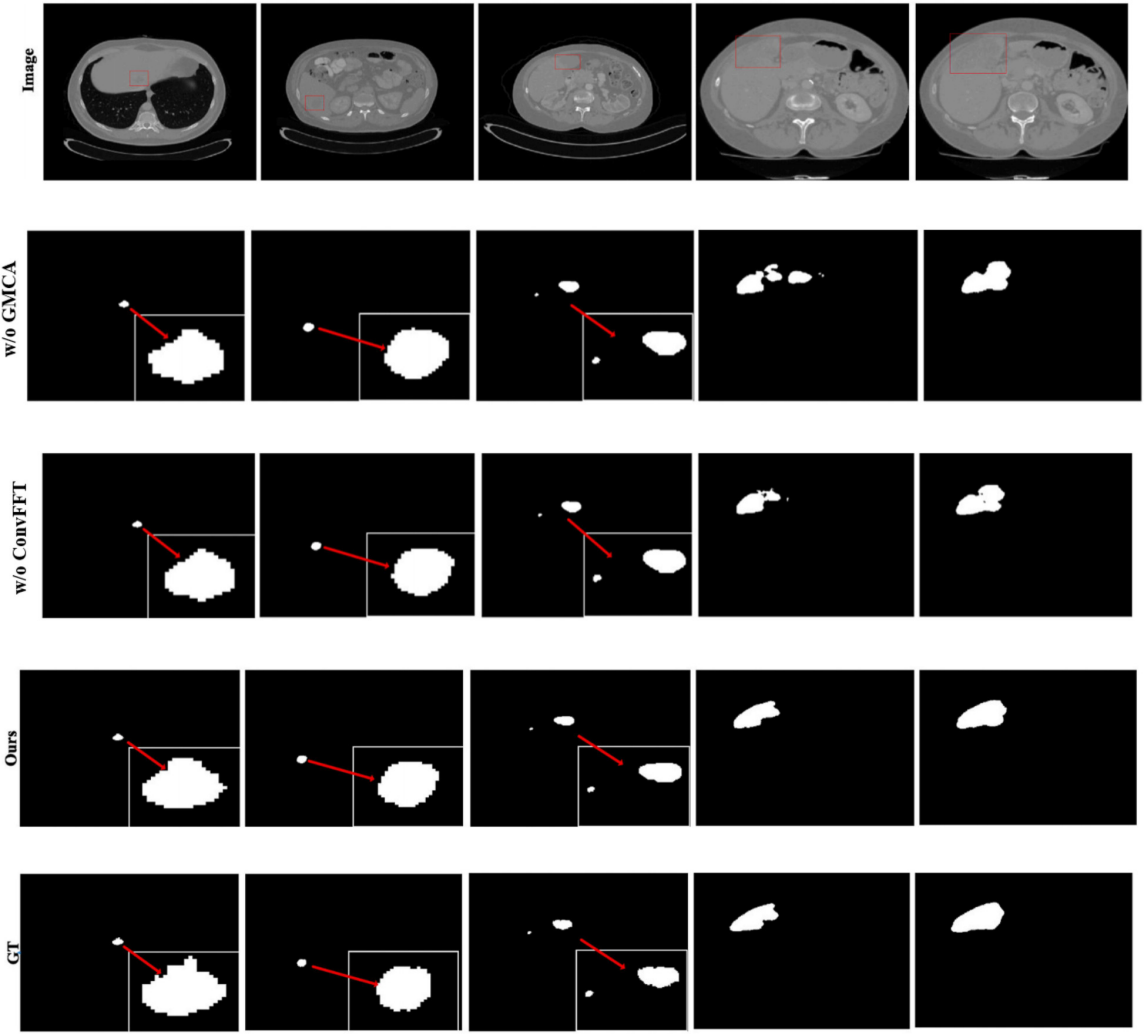
Tumor segmentation results of w/o ConvFFT and w/o GMCA.

Furthermore, Figure 8 presents the visual segmentation images of the two experimental groups with significant result fluctuations: “without ConvFFT module (w/o ConvFFT)” and “without GMCA module (w/o GMCA)”. When the ConvFFT (Convolutional Fast Fourier Transform) module is removed, the FSMF (Fourier Spectral-learning Multi-scale Fusion) branch loses its ability to extract frequency-domain features and can only rely on traditional convolutional layers to process spatial-domain features. At this point, the network's ability to represent tumor details (such as the edges of small tumors and heterogeneous regions) decreases significantly: on one hand, spatial-domain convolution struggles to effectively capture long-term dependencies between pixels, leading to reduced consistency in tumor feature extraction across different cases; on the other hand, the brightness differences of tumors and information about subtle structures (such as tiny lesions) in CT images cannot be enhanced through the frequency domain, making the model more sensitive to minor fluctuations in data distribution.

When the GMCA (Group Multi-Head Cross-Attention Aggregation) module is removed, the network loses the collaborative fusion mechanism for multi-branch features: on one hand, the spectral features from the FSMF branch and the spatial features from the MAHA (Multi-axis Aggregation Hadamard Attention) branch cannot interact effectively, resulting in the model being unable to simultaneously consider both the global distribution (spectral information) and local details (spatial information) of tumors. Differences in tumor size and shape across different cases directly cause feature matching deviations; on the other hand, the decoding process lacks the feature enhancement brought by “multi-scale dilated convolution”, leading to a decline in the localization accuracy of small tumors and irregularly shaped tumors. Over-segmentation or under-segmentation may occur in some cases, which further amplifies the fluctuations in the results.

**Figure 9:**
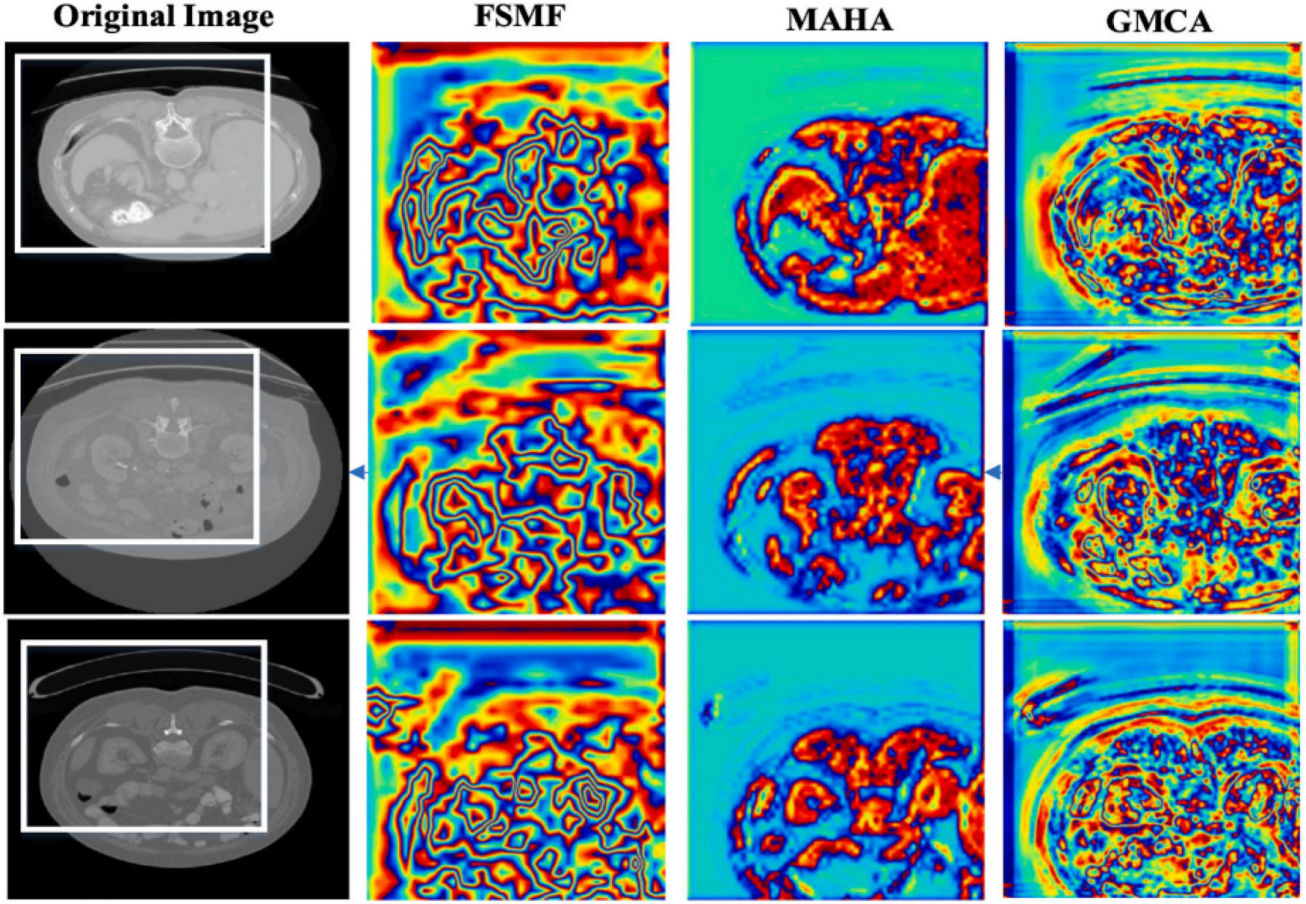
Feature maps form different modules.

In additional, we also show feature maps form different modules in Figure 9. The feature maps of FSMF module exhibit complex textures and edge structures, indicating that this module captures detailed contours (such as lesion edges and tissue textures) of the liver and tumors through Fourier spectral learning and multi-scale fusion. The highlighted regions in the feature maps in MAHA module are concentrated in the spatial areas of the liver and tumors, reflecting that its multi-axis Hadamard attention mechanism enhances discriminative spatial features and can effectively localize pathological regions. The feature maps of GMCA module integrate information from multiple branches, presenting more global inter-pixel dependencies. This shows that its Group Multi-Head Cross-Attention Aggregation module can establish long-range pixel correlations and improve the integrity of features.

#### 4.4.2 Influence of tumor size

To further evaluate the size-dependent performance of DGA-Net, we conducted a lesion-level sensitivity analysis on LiTS2017 by stratifying tumors into three size categories based on the maximum diameter in CT volumes: small tumors (<2 cm), medium tumors (2–5 cm), and large tumors (>5 cm). This stratification aligns with clinical standards for liver tumor classification and addresses the size-dependent variability in segmentation performance. Lesion size was measured manually using the ground-truth annotations to ensure consistency. Only lesions with clear boundaries in the ground truth were included to avoid measurement bias. We adopted DPC as core metrics. Results are presented in Table 5, with comparisons to SOTA models.

**Table 5:** Results of influence of tumor size.

Method	Small tumors (<2 cm)	Medium Tumors (2–5 cm)	Large tumors (>5 cm)
UNet (21)	0.4499 (0.0614) [0.2999, 0.5999]	0.4788 (0.0665) [0.3158, 0.6418]	0.4998 (0.0648) [0.3408, 0.6588]
UNet++ (22)	0.4772 (0.0527) [0.3482, 0.6062]	0.5049 (0.0687) [0.3369, 0.6729]	0.5324 (0.0611) [0.3834, 0.6814]
RA-UNet (23)	0.5572 (0.0438) [0.4502, 0.6642]	0.5874 (0.0522) [0.4594, 0.7154]	0.6154 (0.0427) [0.5104, 0.7204]
nnUNet (24)	0.5972 (0.0687) [0.4292, 0.7652]	0.6232 (0.0487) [0.5042, 0.7422]	0.6542 (0.0511) [0.5292, 0.7792]
EGE-UNet (25)	0.4574 (0.0627) [0.3044, 0.6104]	0.4785 (0.0511) [0.3535, 0.6035]	0.5007 (0.0614) [0.3507, 0.6507]
PA-Net (26)	0.5872 (0.0455) [0.4762, 0.6982]	0.6177 (0.0589) [0.4737, 0.7617]	0.6385 (0.0587) [0.4945, 0.7825]
AGCAF-Net (27)	0.6075 (0.0642) [0.4505, 0.7645]	0.6247 (0.0641) [0.4677, 0.7817]	0.6677 (0.0444) [0.5587, 0.7767]
PVTFormer (28)	0.6252 (0.0701) [0.4532, 0.7972]	0.6487 (0.0587) [0.5047, 0.7927]	0.6742 (0.0428) [0.5692, 0.7792]
**Ours**	0.6352 (0.0521) [0.5082, 0.7622]	0.6784 (0.0484) [0.5604, 0.7964]	0.7152 (0.0387) [0.6202, 0.8102]

All models perform better on larger tumors (consistent with intuition, as small tumors have fewer pixels and weaker contrast). DGA-Net (Ours) outperforms baselines across all size strata, with the most substantial improvement on small tumors. This is attributed to the FSMF branch (capturing fine-grained spectral details) and GMCA module (fusing multi-scale features)—critical for detecting small, low-contrast lesions.

#### 4.4.3 Influence of Gaussian noise on tumors

In this section, we load the images and corresponding labels of the LiTS2017 dataset. In the data preprocessing stage, we add Gaussian noise to the images (35). We only added Gaussian noise to the images, with all other experimental settings remaining unchanged. Table 6 presents the experimental results on the LiTS2017 dataset with manually added Gaussian noise. It compares various methods (with different modules removed, like w/o FSMF, w/o ConvFFT, etc.) and the full DGA-Net (Ours) across metrics: DPC, DG, VOE, RAVD, and ASSD. The full DGA-Net (Ours) achieves the highest DPC (0.6799) and DG (0.7947), and the lowest VOE (0.4485), RAVD (0.4001), and ASSD (11.68). Other methods with individual modules removed show worse performance in these metrics, indicating that the integrated design of the full DGA-Net (Ours) method (with all modules working together) is more robust to Gaussian noise, enabling better tumor segmentation under noisy conditions. The MAHA module is capable of filtering noise and artifacts through dynamic adjustment of multi-axis attention weights and grouped aggregation of features.

**Table 6:** Results of influence of Gaussian noise on tumors.

Method	DPC	DG	VOE	RAVD	ASSD
w/o FSMF	0.6507 (0.0289) [0.5800, 0.7215]	0.7854 (0.0217) [0.7323, 0.8385]	0.4527 (0.0198) [0.4043, 0.5011]	0.7884 (0.0310) [0.7126, 0.8642]	18.19 (0.18) [17.750, 18.630]
w/o ConvFFT	0.6381 (0.0295) [0.5657, 0.7105]	0.5915 (0.0221) [0.5375, 0.6455]	0.5222 (0.0207) [0.4715, 0.5729]	0.5078 (0.0307) [0.4328, 0.5828]	14.74 (0.19) [14.276, 15.204]
w/o GMCA	0.6012 (0.0299) [0.5279, 0.6745]	0.7285 (0.0226) [0.6734, 0.7836]	0.5425 (0.0204) [0.4925, 0.5925]	0.6012 (0.0309) [0.3625, 0.5225]	24.17 (0.18) [23.730, 24.610]
w/o MAHA	0.6383 (0.0297) [0.5654, 0.7112]	0.7519 (0.0222) [0.6974, 0.8064]	0.4897 (0.0189) [0.4435, 0.5360]	0.4425 (0.0326) [0.4411, 0.6007]	13.09 (0.25) [12.476, 13.704)]
w/o One-Branch	0.6509 (0.0291) [0.5796, 0.7221]	0.6614 (0.0214) [0.6091, 0.7137]	0.4839 (0.0197) [0.4358, 0.5320]	0.5209 (0.0325) [0.4081, 0.5637]	26.09 (0.23) [25.526, 26.654]
w/o Add (concat)	0.6639 (0.0284) [0.5944, 0.7331]	0.7107 (0.0215) [0.6581, 0.7633]	0.4703 (0.0194) [0.4231, 0.5175]	0.4859 (0.0317) [0.4081, 0.5637]	17.01 (0.20) [16.522, 17.498]
**Ours**	**0.6799 (0.0298)** [0.6068, 0.7532]	**0.7947 (0.0219)** [0.7411, 0.8483]	**0.4485 (0.0198)** [0.4001, 0.4969]	**0.4001 (0.0318)** [0.3221, 0.4781]	**11.68(0.21)** [11.166, 12.194]

Bold values represent the best mean value.

### 4.5 Cross-dataset evaluation experiments

To further verify the effectiveness of the proposed DGA-Net model, this chapter conducts transfer experiments on multiple datasets, including the 3DIRCADb dataset. Similar to the LiTS2017 dataset, the 3DIRCADb dataset also consists of CT images of liver tumors. Therefore, the model trained on the LiTS2017 dataset is directly used for its testing and evaluation. All experimental models still maintain the same experimental conditions and common hyperparameter settings. To ensure the reliability of the experiment, we repeated each group of experiments three times under the same conditions and took the average value. Among them, the values in parentheses are standard deviations, and those in square brackets are 95% confidence intervals (95%CI).

Tables 7, 8 respectively show the results of several evaluation indicators of 8 models on the 3DIRCADb dataset, including five indicators: DPC, DG, VOE, RAVD, and ASSD. We also show paired two-tailed t-tests (*α*=0.05) with DGA-Net(Ours) in tumors segmentation experiment, and the critical value of |*t*| is 4.303. In the liver segmentation experiment on the 3DIRCADb dataset, the DPC value of DGA-Net is 0.9503 (0.0079), the DG value is 0.9471 (0.0061), the VOE value is 0.0930 (0.0045), the RAVD value is 0.0350 (0.0037), and the ASSD value is 2.65 (0.77). Compared with other models, DGA-Net performs well in multiple metrics. For example, in the DPC metric, it is significantly higher than UNet's 0.9196 (0.0134), indicating that its segmentation results have better consistency with the ground truth. Its VOE value is lower than that of most other models, meaning that the volume error of its segmentation results is smaller. Overall, DGA-Net ranks first in 4 out of the 5 metrics for liver segmentation. This indicates that in the liver segmentation task, the model is superior to comparison models such as UNet and UNet++ in both the accuracy of overall liver recognition and the mastery of segmentation precision and details, demonstrating strong liver segmentation capability.

**Table 7:** Liver results of each model on the 3DIRCADb.

Method	DPC	DG	VOE	RAVD	ASSD
UNet (21)	0.9196 (0.0134) [0.8865,0.9527]	0.9136 (0.0115) [0.8852,0.9420]	0.1440 (0.0092) [0.1212,0.1668]	0.0825 (0.0071) [0.0649,0.1001]	11.54 (1.89) [6.86,16.21]
UNet++ (22)	0.9374 (0.0115) [0.9090,0.9658]	0.9332 (0.0102) [0.9080,0.9584]	0.1160 (0.0089) [0.0940,0.1380]	0.0599 (0.0062) [0.0446,0.0752]	9.74 (1.56) [5.87,13.60]
RA-UNet (23)	0.9295 (0.0095) [0.9060,0.9530]	0.9231 (0.0058) [0.9088,0.9374]	0.1230 (0.0053) [0.1099,0.1361]	0.0706 (0.0059) [0.0560,0.0852]	6.30 (1.44) [2.73,9.86]
nnUNet (24)	0.9460 (0.0104) [0.9203,0.9717]	**0.9480 (0.0065)** [0.9319,0.9641]	0.1007 (0.0065) [0.0846,0.1168]	0.0485 (0.0043) [0.0379,0.0601]	3.23 (0.85) [1.13,5.32]
EGE-UNet (25)	0.8932 (0.0127) [0.8618,0.9246]	0.8913 (0.0087) [0.8698,0.9128]	0.1892 (0.0041) [0.1791,0.1993]	0.1145 (0.0039) [0.1049,0.1241]	9.68 (1.32) [6.41,12.94]
PA-Net (26)	0.9451 (0.0069) [0.9280,0.9622]	0.9409 (0.0094) [0.9177,0.9641]	0.1029 (0.0049) [0.0908,0.1150]	0.0466 (0.0046) [0.0352,0.0580]	6.08 (1.05) [3.48,8.67]
AGCAF-Net (27)	0.9428 (0.0092) [0.9200,0.9656]	0.9388 (0.0083) [0.9183,0.9593]	0.1001 (0.0054) [0.0868,0.1134]	0.0424 (0.0051) [0.0298,0.0550]	4.20 (0.89) [2.00,6.39]
PVTFormer (28)	0.9479 (0.0084) [0.9271,0.9687]	0.9408 (0.0078) [0.9215,0.9601]	0.1059 (0.0068) [0.0891,0.1227]	0.0409 (0.0048) [0.0290,0.0528]	3.85 (0.84) [1.77,5.92]
**Ours**	**0.9503 (0.0079)** [0.9307,0.9699]	0.9471 (0.0061) [0.9320,0.9622]	**0.0930 (0.0045)** [0.0819,0.1041]	**0.0350 (0.0037)** [0.0259,0.0441]	**2.65(0.77)** [0.74,4.55]

Bold values represent the best mean value.

**Table 8:** Tumor results of each model on 3DIRCADb.

Method	DPC	DG	VOE	RAVD	ASSD
UNet (21)	0.6312 (0.0159) [0.5919,0.6705]	0.7407 (0.0121) [0.7107,0.7707]	0.4840 (0.0085) [0.4630,0.5050]	1.3807 (0.1769) [0.9429,1.8185]	19.40 (1.87) [14.77,24.02]
	*t* = 11.08, S	*t* = 11.47, S	*t* = −20.75, S	*t* = −10.13, S	*t* = −9.09, S
UNet++ (22)	0.7006 (0.0141) [0.6657,0.7355]	0.7683 (0.0115) [0.7399,0.7967]	0.4123 (0.0074) [0.3940,0.4306]	1.2229 (0.1687) [0.8050,1.6408]	17.39 (1.67) [13.25,21.52]
	*t* = 4.15, S	*t* = 7.79, S	*t* = −5.56, S	*t* = −9.09, S	*t* = −7.79, S
RA-UNet (23)	0.7127 (0.0132) [0.6801,0.7453]	0.8099 (0.0104) [0.7842,0.8356]	0.4051 (0.0051) [0.3925,0.4177]	0.4377 (0.0084) [0.4169,0.4585]	13.34 (1.54) [9.52,17.15]
	*t* = 2.83, NS	*t* = 1.69, NS	*t* = −7.3, S	*t* = −18.72, S	*t* = −4.06, NS
nnUNet (24)	0.6946 (0.0107) [0.6681,0.7211]	0.7625 (0.0099) [0.7380,0.7870]	0.4443 (0.0048) [0.4324,0.4562]	0.4045 (0.0287) [0.3335,0.4755]	13.08 (1.97) [8.19,17.96]
	*t* = 6.41, S	*t* = 9.99, S	*t* = −22.94, S	*t* = −3.49, S	*t* = −2.92, S
EGE-UNet (25)	0.5395 (0.0149) [0.5027,0.5763]	0.6498 (0.0092) [0.6270,0.6726]	0.6268 (0.0054) [0.6135,0.6401]	1.2104 (0.1543) [0.8285,1.5923]	27.09 (2.56) [20.75,33.42]
	*t* = 22.64, S	*t* = 32.73, S	*t* = −78.97, S	*t* = −9.74, S	*t* = −11.85, S
PA-Net (26)	0.7163 (0.0154) [0.6782,0.7544]	0.7979 (0.0095) [0.7744,0.8214]	0.4064 (0.0059) [0.3918,0.4210]	0.5744 (0.0095) [0.5509,0.5979]	12.38 (1.42) [8.86,15.89]
	*t* = 1.99, NS	*t* = 4.19, S	*t* = −7.09, S	*t* = −43.37, S	*t* = −3.22, NS
AGCAF-Net (27)	0.6841 (0.0134) [0.6510,0.7172]	0.8100 (0.0102) [0.7848,0.8352]	0.4450 (0.0055) [0.4314,0.4586]	0.4000 (0.0079) [0.3804,0.4196]	11.20 (1.12) [8.42,13.97]
	*t* = 6.47, S	*t* = 2.36, NS	*t* = −19.69, S	*t* = −11.94, S	*t* = −2.22, NS
PVTFormer (28)	0.6878 (0.0121) [0.6578,0.7178]	0.8120 (0.0091) [0.7895,0.8345]	0.4420 (0.0051) [0.4294,0.4546]	0.3950 (0.0074) [0.3767,0.4133]	11.00 (1.04) [8.42,13.57]
	*t* = 6.7, S	*t* = 1.57, NS	*t* = −19.97, S	*t* = −11.6, S	*t* = −2.02, NS
**Ours**	**0.7343 (0.0101)** [0.7093,0.7593]	**0.8202 (0.0098)** [0.7960,0.8444]	**0.3829 (0.0047)** [0.3713,0.3945]	**0.3466 (0.0067)** [0.3300,0.3632]	**9.77 (0.89)** [7.57,11.96]

Bold values represent the best mean value.

DGA-Net performs even more prominently in tumor segmentation. Its DPC value is 0.7343 (0.0101), DG value is 0.8202 (0.0098), VOE value is 0.3829 (0.0047), RAVD value is 0.3466 (0.0067), and ASSD value is 9.77 (0.89). Compared with other models, it leads by a significant margin in all metrics. In particular, the two key metrics of DPC and DG are both nearly 2% higher than those of the second-place model, which indicates that this model can identify tumor regions more accurately in tumor segmentation, and its segmentation results have a higher similarity to the real situation. Compared with other models, when dealing with complex tumor conditions, DGA-Net can more accurately capture the tumor boundary information, and its segmentation results are more continuous and complete, which further verifies its excellent performance in tumor segmentation tasks.

**Figure 10:**
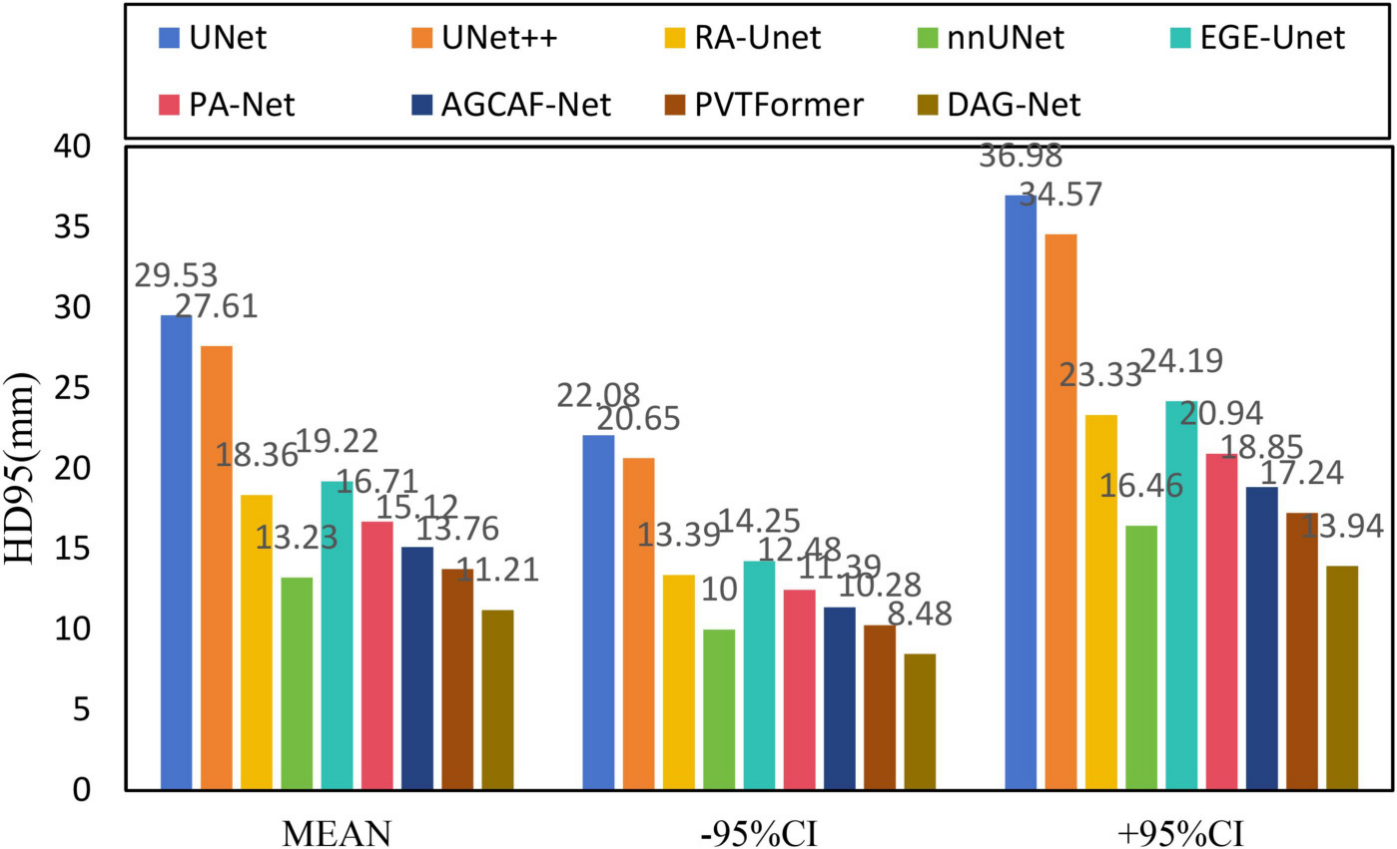
HD95 of each model on 3DIRCADb.

Figure 10 shows the experimental results of HD95 on 3DIRCADb. The experimental environment and settings are consistent, and each model is run three times, with the average value taken. And we report 95%CI. DGA-Net has the lowest HD95, indicating that the spatial difference between the tumor segmentation result and the ground truth is the smallest, and the segmentation accuracy is the highest. In contrast, the Lits2017 dataset exhibits more complex and diverse image features, leading to higher segmentation difficulty.

**Figure 11:**
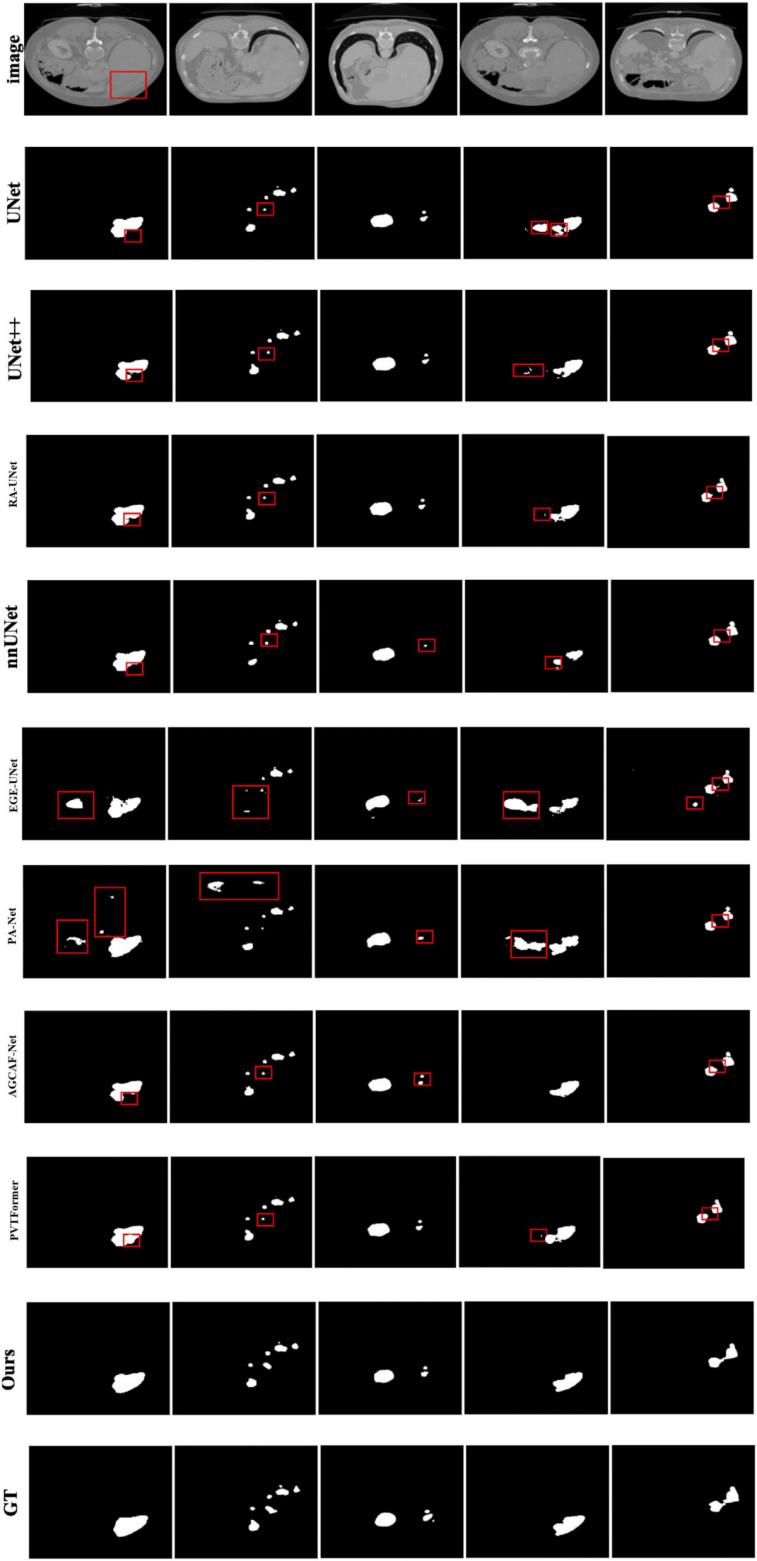
Tumor segmentation results of each model on 3DIRCADb.

Since the liver segmentation results are generally high and the differences between them are small, this section only shows some styles of tumor segmentation results to intuitively reflect the segmentation performance of each model, as shown in Figure 11. It can be clearly seen from the figure that the DGA-Net model also has excellent segmentation performance for tumors with irregular shapes, sizes, and different quantities.

**Figure 12:**
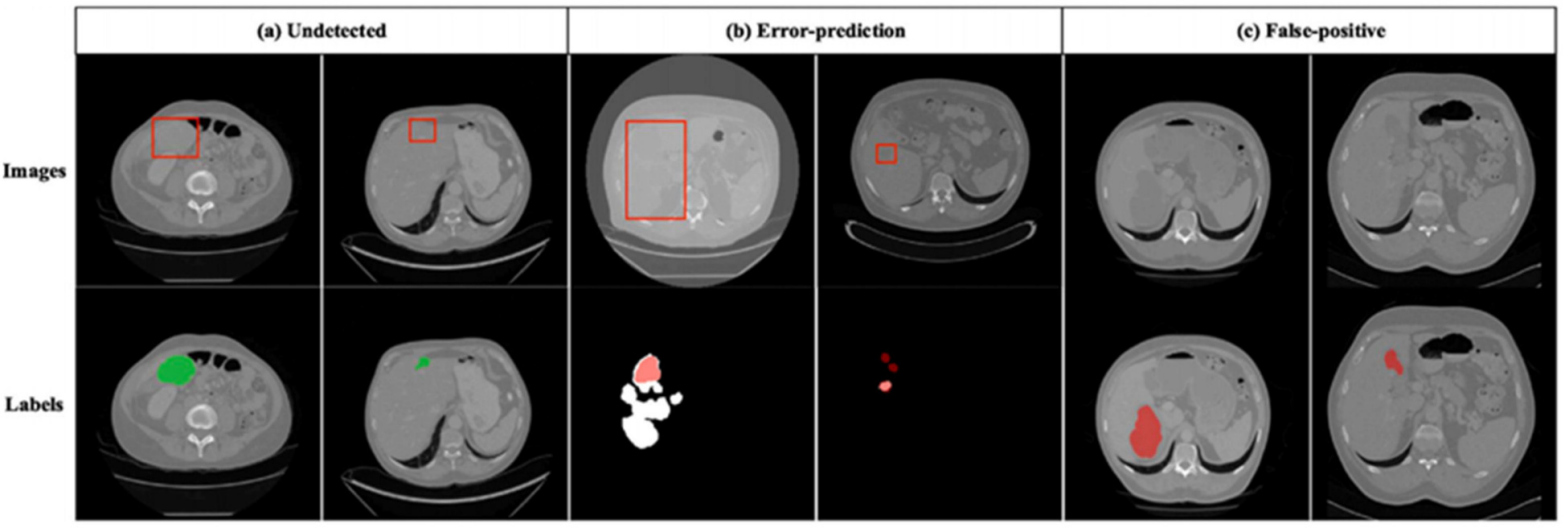
Incorrect segmentation results on 3DIRCADb. **(a)** Undetected. **(b)** Error-prediction. **(c)** False-positive.

Figure 12 shows incorrect segmentation results on 3DIRCADb using DGA-Net. Figure 12a is non-complete segmentation result for the undetected liver tumor. Figure 12b is the error predicted result for liver tumor segmentation. Figure 12c is the false positive segmentation result. These error cases intuitively present the limitations of the model and provide directions for subsequent improvements: For undetected cases, the feature extraction module for small lesions can be optimized. For error-prediction cases, multi-scale feature fusion or the attention mechanism needs to be improved to enhance the accuracy of boundary recognition. For false-positive cases, the feature discrimination ability between normal tissues and tumors should be strengthened to reduce misjudgments.

### 4.6 Analysis and discussion

Combining quantitative and qualitative analyses, the limitations of various comparative methods in liver tumor segmentation are summarized as follows: U-Net (21)/UNet++ (22) show “zigzag” discontinuities at tumor boundaries and high missed detection rates for small tumors due to relying solely on skip connections for feature fusion, lacking global context and long-term pixel dependence modeling. nnUNet (24) performs well in liver segmentation but poorly adapts to small or irregular tumors, as its adaptive architecture focuses more on whole-organ scale adaptation, lacks spectrum or attention mechanisms, and has large parameters. EGEU-Net (25), a lightweight model with few parameters, has limited feature extraction capability, leading to incomplete segmentation in complex tumor regions like multi-lesion fusion. RA-UNet (23)/PA-Net (26)/AGCAF-Net (27)/PVTFormer (28), despite introducing attention mechanisms, only focus on local feature interaction, lack multi-axis global feature aggregation (e.g., MAHA's cross-channel-spatial modeling), and have large parameters, resulting in deficiencies in distinguishing tumors from adjacent structures.

For DGA-Net, the FSMF module enhances the boundary discriminability between tumors and normal tissues through Fourier spectrum features, thereby improving the detection of small tumors; the MAHA module uses Hadamard product fusion to achieve efficient global-local feature interaction with fewer parameters, reducing computational costs; the GMCA module integrates multi-branch features to improve tumor localization accuracy (especially for irregular tumors) and assists in surgical planning by optimizing boundaries.

Although this study has ensured the fairness of baseline model comparisons through a unified training process, there remain potential residual biases, primarily stemming from inherent differences in model architectures: First, differences in computational constraints between 2D and 3D models. The DGA-Net in this study is a 2D architecture, and experiments adopted a unified 2D training paradigm to match its input format. This prevented 3D baselines such as nnUNet from fully leveraging their advantage in utilizing cross-slice spatial information, which may introduce a slight bias in performance comparisons. Second, trade-off biases related to memory and computational costs. The original design of 3D baselines like RA-UNet relies on larger memory capacity. Under the constraint of a unified batch size (batch size = 4) in this study, their training did not reach an optimal performance state. This creates a discrepancy in environmental adaptability compared to the lightweight 2D design of DGA-Net. Third, architectural adaptability biases. The self-attention mechanism of Transformer-based baselines such as PVTFormer exhibits lower computational efficiency on 2D slices than in 3D scenarios. Their inherent advantages, such as global context modeling, were not fully realized within the unified training framework, which may affect the absolute validity of performance comparisons.

Future research directions will focus on the clinical translation and performance optimization of the model: on one hand, we will conduct multi-center clinical cooperation to collect clinical CT image cohorts from different hospitals and scanning equipment, as well as patients’ pathological information, treatment plans, and prognostic data. Combined with confidence intervals and patient-level clinical indicators such as tumor detection sensitivity and clinical staging consistency, we will systematically verify the clinical utility of the model. Meanwhile, we will perform robustness analysis targeting the heterogeneity of clinical data, evaluate the model's performance under different scanning parameters, lesion sizes/locations/pathological types to optimize its adaptability to clinical data, and explore the construction of a two-stage segmentation framework and a multi-modal fusion model to improve the segmentation accuracy of small and irregular lesions; on the other hand, to address the limitations of DGA-Net's 2D nature in utilizing inter-slice information and segmenting cross-slice tumors, we will explore extending it to a 3D architecture, model inter-slice spatial correlations and global structures through 3D convolutions or spatiotemporal attention mechanisms, research multi-scale feature fusion strategies that integrate contextual information from adjacent slices to enhance the recognition accuracy of small tumors and edge regions, and optimize the computational efficiency of the 3D model through lightweight design or dynamic inference mechanisms on the premise of ensuring segmentation performance, achieving a balance between accuracy and cost and laying a more solid foundation for the clinical application of the model (36).

## 5 Conclusions

To address the issues of missing dependencies between pixels and high model parameter requirements, we propose the DGA-Net model. This model consists of a dual-branch encoder (FSMF module and MAHA module) and a decoder with a GMCA module. The FSMF module learns amplitude and phase information to capture rich features; the MAHA module enhances discriminative features while reducing costs; and the GMCA module improves positioning capability and establishes long-term dependencies between pixels. Experimental results show that this method outperforms advanced methods on the LiTS2017 dataset, with the segmentation accuracy (DPC) of the liver and tumors reaching 95.23% and 69.51% respectively. It also performs excellently on other datasets, demonstrating good generalization ability. DGA-Net boasts excellent liver/tumor segmentation accuracy and efficient inference, holding great promise to transform clinical radiological workflows. This data support is expected to optimize preoperative planning and enhance the objectivity of postoperative efficacy evaluation, laying a solid foundation for more standardized, efficient liver tumor diagnosis and treatment.

## Data Availability

Publicly available datasets were analyzed in this study. This data can be found here: https://www.kaggle.com/andrewmvd/liver-tumor-segmentation, http://m6z.cn/6x5OSn.

## Footnotes

1
https://github.com/ygp1234/DGA-Net

2
https://www.kaggle.com/andrewmvd/liver-tumor-segmentation

3
http://m6z.cn/6x5OSn
